# Metabolomic profile of prostate cancer-specific survival among 1812 Finnish men

**DOI:** 10.1186/s12916-022-02561-4

**Published:** 2022-10-25

**Authors:** Jiaqi Huang, Bin Zhao, Stephanie J. Weinstein, Demetrius Albanes, Alison M. Mondul

**Affiliations:** 1 National Clinical Research Center for Metabolic Diseases, Key Laboratory of Diabetes Immunology, Ministry of Education, and Department of Metabolism and Endocrinology, The Second Xiangya Hospital of Central South University, Changsha, 410011 Hunan China; 2 Division of Cancer Epidemiology and Genetics, National Cancer Institute, National Institutes of Health, Bethesda, MD 20892 USA; 3 Department of Epidemiology, University of Michigan School of Public Health, Ann Arbor, MI 48109 USA

**Keywords:** Metabolomic profile, Prostate cancer survival, Choline, Glutamate, Gamma-glutamyl amino acids, Fibrinopeptides, Endocannabinoid, Redox metabolites

## Abstract

**Background:**

Abnormal metabolism and perturbations in metabolic pathways play significant roles in the development and progression of prostate cancer; however, comprehensive metabolomic analyses of human data are lacking and needed to elucidate the interrelationships.

**Methods:**

We examined the serum metabolome in relation to prostate cancer survival in a cohort of 1812 cases in the Alpha-Tocopherol, Beta-Carotene Cancer Prevention (ATBC) Study. Using an ultrahigh-performance LC-MS/MS platform, we identified 961 known metabolites in prospectively collected serum. Median survival time from diagnosis to prostate cancer-specific death (*N*=472) was 6.6 years (interquartile range=2.9–11.1 years). Cox proportional hazards regression models estimated hazard ratios and 95% confidence intervals of the associations between the serum metabolites (in quartiles) and prostate cancer death, adjusted for age at baseline and diagnosis, disease stage, and Gleason sum. In order to calculate risk scores, we first randomly divided the metabolomic data into a discovery set (70%) and validated in a replication set (30%).

**Results:**

Overall, 49 metabolites were associated with prostate cancer survival after Bonferroni correction. Notably, higher levels of the phospholipid choline, amino acid glutamate, long-chain polyunsaturated fatty acid (n6) arachidonate (20:4n6), and glutamyl amino acids gamma-glutamylglutamate, gamma-glutamylglycine, and gamma-glutamylleucine were associated with increased risk of prostate cancer-specific mortality (fourth versus first quartile HRs=2.07–2.14; *P*-values <5.2×10^−5^). By contrast, the ascorbate/aldarate metabolite oxalate, xenobiotics S-carboxymethyl-L-cysteine, fibrinogen cleavage peptides ADpSGEGDFXAEGGGVR and fibrinopeptide B (1-12) were related to reduced disease-specific mortality (fourth versus first quartile HRs=0.82–0.84; *P*-value <5.2×10^−5^). Further adjustment for years from blood collection to cancer diagnosis, body mass index, smoking intensity and duration, and serum total and high-density lipoprotein cholesterol did not alter the results. Participants with a higher metabolic score based on the discovery set had an elevated risk of prostate cancer-specific mortality in the replication set (fourth versus first quartile, HR=3.9, *P*-value for trend<0.0001).

**Conclusions:**

The metabolic traits identified in this study, including for choline, glutamate, arachidonate, gamma-glutamyl amino acids, fibrinopeptides, and endocannabinoid and redox pathways and their composite risk score, corroborate our previous analysis of fatal prostate cancer and provide novel insights and potential leads regarding the molecular basis of prostate cancer progression and mortality.

**Supplementary Information:**

The online version contains supplementary material available at 10.1186/s12916-022-02561-4.

## Background

With a global estimate of 1.4 million new cases and 375,000 deaths, prostate cancer is the second most commonly diagnosed malignancy and the fifth most frequent cause of cancer death among men in 2020, contributing to a considerable health burden among men both in the USA and worldwide [1]. Despite this reality, prostate cancer etiology and effective means for its prevention remain incompletely understood. The well-recognized risk factors for prostate cancer include older age, African ancestry, family history of prostate cancer, and low-penetrant genetic variants, none of which are modifiable [1]. It is well-accepted that many prostate cancer cases, particularly those diagnosed through prostate-specific antigen (PSA) screening, have microscopic foci of limited (i.e., non-life-threatening) clinical relevance, and research suggests that more aggressive and fatal prostate malignancies have etiologies distinct from indolent disease [2–4]. Thus, there is a need for further study of prostate cancer survival.

A poor understanding of the etiology of prostate cancer survival limits our ability to make meaningful recommendations regarding prevention and effective risk stratification for men diagnosed with prostate cancer. Alterations in metabolism and perturbations in metabolic pathways can play significant roles in the development and progression of prostate cancer; however, substantial human data are needed to elucidate these associations [5, 6]. Recent advances of high-throughput metabolomics approaches enable the systematic measurement of a broad spectrum of small compounds, and the unique metabolic profiles can reflect an integrated final molecular phenotype of endogenous and exogenous exposure, including influence from gene-environment interactions, that will facilitate the identification of novel risk factors and metabolic pathways associated with disease progression [7–9].

Metabolomic analyses of nested case-control sets within three major cohorts have explored associations between pre-diagnostic levels of circulating metabolites and risk of prostate cancer [10–14]. Associations between urine and tissue metabolites and risk of prostate cancer have also been reported in some clinical studies [15, 16]. To our knowledge, only two untargeted metabolomic analyses have been conducted examining fatal prostate cancer specifically. One investigation included 523 prostate cancer cases in the Alpha-Tocopherol, Beta-Carotene Cancer Prevention (ATBC) Study [13] and showed risk associations with glutathione-reactive oxygen species (ROS) metabolites including thioproline, cysteine, and cystine, while another smaller study found endocannabinoid N-oleoyl taurine and sterol/steroids metabolites related to prostate cancer survival [17]. Thus, there remains a substantial research gap for the investigation of circulating metabolite profiles in relation to prostate cancer survival, that urgently warrants investigation.

We conducted a prospective untargeted metabolomic analysis with the aim to identify pre-diagnostic serum metabolic profiles and metabolic pathways that are associated with survival among 1812 prostate cancer patients within the ATBC Study. We hypothesize that unique metabolomic profiles would be associated with prostate cancer-specific survival.

## Methods

### Study population

The ATBC Study was a double-blind, 2×2 factorial, randomized, placebo-controlled trial that enrolled participants from 1985 to 1988, and the primary aim of this intervention study was to test whether supplementation of alpha-tocopherol and beta-carotene could reduce cancer incidence, especially for lung cancer. The ATBC Study enrolled 29,133 Finnish male smokers, who were aged 50 to 69 years old from southwest Finland, and the eligible participants were assigned into 4 groups to receive the supplement daily (alpha-tocopherol, beta-carotene, both, or placebo) for 5–8 years until the end of the intervention (April 30, 1993). At enrollment, participants completed questionnaires that included detailed information on lifestyle risk factors and a separate validated food frequency questionnaire. Height, weight, heart rate, and blood pressure were measured, and overnight fasting blood samples were collected by research nurses. The blood samples were then aliquoted and stored at −70 °C until assayed.

### Outcome assessment

Via linkage to the Finnish Cancer Registry by using the International Classification of Disease (ICD-9) code of 185, we identified 1812 incident prostate cancer cases diagnosed through December 31, 2012, with available non-thawed baseline serum and non-missing cancer stage data. We used the underlying cause of death from the Statistics Finland Death Registry to identify prostate cancer-specific mortality, based on the code 185 of ICD-9 and C61 of ICD-10. In the present study, we found 472 prostate cancer deaths that occurred during the observational follow-up period through December 31, 2016. The survival time was computed from the date of prostate cancer diagnosis to the date of prostate cancer-specific mortality, or to the censor date (date of death due to other causes or December 31, 2016), whichever occurred first.

### Metabolomic profiling assays

Serum metabolite profiling was conducted on a high-resolution accurate mass (HRAM) platform, ultrahigh-performance liquid chromatography/tandem mass spectrometry (LC-MS/MS) at Metabolon Inc. We measured 1195 metabolites, of which 234 were excluded due to being unidentified metabolites or metabolites for which more than 90% of subjects had values below the limit of detection, leaving 961 metabolites in the final analysis. For those metabolites remaining after these exclusions were applied, undetectable values were assigned to one-half minimum value of the metabolite. Based on biochemical information from the Kyoto Encyclopedia of Genes and Genomics (KEGG) database, and the Human Metabolome Database, identified known metabolites were classified into different chemical classes: amino acids or amino acid derivatives (further referred to as “amino acids”), carbohydrates, cofactors and vitamins, energy biochemicals, lipids, nucleotides, peptides or xenobiotics. In order to assess the reliability and reproducibility of the biochemical data, each metabolite’s intraclass correlation coefficient (ICC) and coefficient of variation (CV) were computed based on blinded quality control samples embedded in each batch (6%). The median ICC and CV for measured metabolites were 0.95 (interquartile range=0.87 to 0.98) and 0.12 (interquartile range=0.07 to 0.23), respectively.

Serum levels of alpha-tocopherol and retinol quantified by metabolomic profiling were highly correlated with concentrations originally assayed for the entire cohort at baseline using the reversed-phase isocratic high-performance liquid chromatography (HPLC) method, suggesting excellent validity and reproducibility for the present metabolomic assay platform (alpha-tocopherol: *r*=0.75, *P*-value <10^−8^; retinol, *r*=0.77, *P*-value <10^−8^).

### Statistical analysis

In order to control for batch variation, metabolites were batch-normalized and divided by the batch median value. Then each metabolite was log-transformed and normalized with a mean of 0 and a variance of 1. We used Cox proportional hazards regression models to calculate the hazard ratios (HRs) and 95% confidence intervals (CIs) of the associations between the serum metabolites and prostate cancer death, adjusted for age at baseline, age at prostate cancer diagnosis, disease stage, and Gleason sum, and we modeled standardized metabolites as both categorical (quartiles) and continuous variables (per 1-standard deviation [SD] log-metabolite increment). In a separate Cox proportional regression model, we tested for linear trend by assigning the ordinal value of the quartile and treating this as a continuous variable. To control for multiple hypothesis testing [18], metabolite-risk associations with a Bonferroni corrected threshold (a *P*-value of 5.2×10^−5^ or less, 0.05/961) were considered statistically significant.

In additional analyses, we further adjusted for body mass index (BMI, weight (kg)/height (m)^2^), number of cigarettes smoked daily, years of smoking, total serum cholesterol (mmol/L), serum high-density lipoprotein cholesterol (mmol/L), and calendar year of cancer diagnosis (all as continuous variables). We also performed analyses stratified by time from blood collection to prostate cancer diagnosis (median split), disease stage at diagnosis (stage I–II and stage III–IV) and BMI (<25, 25–<30, and ≥30 kg/m^2^). In the entire dataset, we conducted a forward stepwise Cox proportional hazards regression to determine the set of metabolites that were conditionally independently associated with prostate cancer death, with an alpha-error, the Bonferroni corrected threshold of .05/961 to enter the model (i.e., top 49 signals) and *P*=.05 to remain in the model. For comparison of the ability for different clinical factors and selected metabolites to distinguish between prostate cancer-specific mortality and non-prostate cancer-specific mortality, we utilized adjusted receiver operating characteristics (ROC) and adjusted area under the curve (AUC) analyses. Differences between AUC were tested using the method proposed by DeLong et al. [19],

Pathway analyses evaluated the associations between chemical sub-classes of metabolites and risk of prostate cancer-specific mortality. Due to the correlations between metabolites in the sub-class pathways, we used a parametric bootstrap method to calculate the *P*-value from each pathway level (based on 100,000 permutations), with the assumption that the *Z*-statistics of the component metabolites followed a multivariate normal distribution with a mean of 0 and estimated covariance matrix [20, 21]. For pathways associated with prostate cancer-specific mortality, we conducted principal component analysis (PCA) using the varimax rotation method and defined the “pathway score” by the first generated principal component. Cox proportional hazards regression models were applied to estimate the HRs of prostate cancer-specific mortality for the resultant PCA score.

In order to calculate risk scores, we first randomly divided the metabolomic data into a discovery set (70%) and a replication set (30%). Within the discovery set, we used Cox proportional hazards regression models to select metabolites (Bonferroni corrected threshold, a *P*-value of 5.2×10^−5^ or less, 0.05/961) associated with prostate cancer-specific mortality (number of metabolites=5). We then created a risk score (sum) of these metabolites in the replication set weighted by their coefficients as obtained from the Cox regression models in the discovery set. The metabolite risk score was then normalized and modeled as both quartiles and as a continuous variable (with a mean=0 and SD=1) to estimate the association with prostate cancer-specific mortality, according to the Cox regression model and adjusted for the above-mentioned covariates.

All analyses were conducted using SAS version 9.4 (SAS Institute, Cary, NC), and R version 3.6.1 (R Development Core Team, Vienna, Austria). The reported statistical tests were two-sided.

## Results

Baseline and clinical characteristics of the 1812 prostate cancer cases being studied are presented in Table 1. Through the end of follow-up, we observed 1522 deaths (84.0%) of which 472 died from prostate cancer (26.1% of all cases). Comparing cases who died from prostate cancer to those who did not, the median age at diagnosis was 69.0 years (interquartile range=65.0 to 73.0) and 71.0 years (interquartile range= 68.0 to 75.0), respectively, and the median time from baseline blood collection to cancer diagnosis was 11.0 years (interquartile range=8.0 to 15.0) and 14.0 (interquartile range=11.0 to 18.0), respectively. The cancer stage distribution among cases who died from prostate cancer was 39.4% (cancer stage I or II), 15.3% (locally advanced disease, stage III), and 45.3% (metastatic disease, stage IV), whereas for those who did not die from prostate cancer it was 81.9%, 12.6%, and 5.5%, respectively.

**Table 1:** Selected baseline and clinical characteristics of men with prostate cancer

Characteristic ^**a**^	Died from prostate cancer (***N*** = 472)	Did not die from prostate cancer (***N*** = 1340)
Age at blood collection, years, median (interquartile range)	58.0 (54.0–62.0)	57.0 (53.0–61.0)
Age at cancer diagnosis, years, median (interquartile range)	69.0 (65.0–73.0)	71.0 (68.0–75.0)
Time from baseline to cancer diagnosis, years, median (interquartile range)	11.0 (8.0–15.0)	14.0 (11.0–18.0)
BMI (kg/m^2^)	26.2 (3.6)	26.3 (3.6)
Serum total cholesterol (mmol/L)	6.2 (1.1)	6.2 (1.1)
Serum HDL cholesterol (mmol/L)	1.2 (0.3)	1.2 (0.3)
Serum α-tocopherol (mg/L)	11.8 (3.1)	11.8 (3.1)
Serum β-carotene (μg/L)	228 (205)	225 (178)
Serum retinol (μg/L)	601 (128)	595 (129)
Cigarette per day	19 (8)	20 (9)
Years of cigarette smoking	36 (9)	35 (9)
Diagnosis period, No. (%)		
1986–1994	191 (40.4)	253 (18.9)
1995–2000	165 (35.0)	478 (35.7)
2001–2010	116 (24.6)	609 (45.5)
Cancer stage, No. (%)		
Stage I	73 (15.5)	580 (43.3)
Stage II	113 (23.9)	517 (38.6)
Stage III	72 (15.3)	169 (12.6)
Stage IV	214 (45.3)	74 (5.5)
Gleason score, No. (%)		
1–6	112 (23.7)	698 (52.1)
7	103 (21.8)	282 (21.0)
8–10	120 (25.4)	131 (9.8)
Unknown	137 (29.0)	229 (17.1)
Median survival time for all case patients (years)	5.0 (4.1)	8.3 (5.4)
Median survival time for case patients by stage (years)		
Stage I	7.8 (4.4)	8.3 (5.1)
Stage II	6.4 (3.8)	8.5 (5.7)
Stage III	5.9 (4.3)	9.3 (5.6)
Stage IV	3.1 (3.0)	5.3 (5.0)
Median survival time for case patients by Gleason score (years)		
1–6	6.5 (4.6)	8.8 (5.5)
7	5.0 (3.6)	8.1 (4.8)
8–10	3.8 (3.1)	6.2 (5.0)
Unknown	5.0 (4.5)	8.5 (6.1)

^a^ Values are means (standard deviation) unless otherwise indicated

Cox proportional regression models found that 49 of the 961 identified metabolites were associated with prostate cancer mortality at the Bonferroni corrected *P*-value threshold of 5.2×10^−5^ or less, including 18 peptides, 14 amino acids, 12 lipids, two cofactors/vitamins, and one each of nucleotides, carbohydrates, and xenobiotics (Table 2). The two strongest metabolite associations with prostate cancer death were for the lipid choline (fourth versus first quartile, HR=2.07, 95% CI:1.60–2.69, *P*_trend_ across quartiles=7.2×10^−11^), and the amino acid glutamate (HR=2.31, 95% CI: 1.77–3.02, *P*_trend_=6.7×10^−10^). Other statistically significant positive associations included the following: 11 gamma-glutamyl amino acids (fourth versus first quartile, HRs=1.58–2.14; 1.3×10^−9^≤*P*≤3.1×10^−5^), three endocannabinoids (HRs=1.62–1.69; 3.1×10^−5^≤*P*≤4.6×10^−5^), three dipeptides (HRs=1.83–1.97; 3.0×10^−7^≤*P*≤4.9×10^−6^), two metabolites in the glycine-serine-threonine metabolism pathway (HRs=1.81–1.95; 3.7×10^−8^≤*P*≤3.3×10^−6^), two metabolites in the methionine-cysteine-SAM-taurine pathway (HRs=1.96–2.06; 2.8×10^−8^≤*P*≤2.5×10^−7^), two lysophospholipids (HRs=1.61–1.76; 2.7×10^−6^≤*P*≤2.7×10^−5^), one fibrinogen cleavage peptide, fibrinopeptide B (1-9) (HR=1.80, 95% CI:1.38–2.36; *P*_trend_=4.0×10^−6^), and the glutathione metabolite 5-oxoproline (HR=1.67, 95% CI:1.30–2.16; *P*_trend_=2.3×10^−6^). We also observed significant inverse associations for three fibrinogen cleavage peptides (fourth versus first quartile, HRs=0.54–0.60; 5.1×10^−6^≤*P*≤5.2×10^−5^), the three glutathione metabolites cysteine-glutathione disulfide, Cys-Gly, and oxidized cysteinylglycine (HRs=0.53–0.59; 1.4 ×10^−5^≤*P*≤4.1×10^−5^), and two ascorbate-aldarate metabolites, oxalate and threonate (HRs=0.75 and 0.80; *P*_trend_=3.0×10^−6^ and 3.2×10^−5^, respectively) (Table 2).

**Table 2:** Hazard ratios and 95% confidence intervals for the association between prostate cancer mortality and prediagnostic serum metabolites achieving the Bonferroni corrected threshold based on 1812 prostate cancer cases in the ATBC study ^**a**^

Metabolite and chemical class	Chemical sub-class pathway	Quartile of metabolite				HR (95% CI) per 1-SD	***P***-value for trend ^**b**^
		1	2	3	4		
**Amino acids and amino acid derivatives**		HR	HR (95% CI)	HR (95% CI)	HR (95% CI)		
Aspartate	Alanine and aspartate metabolism	1.00	1.44 (1.07, 1.94)	1.12 (0.83, 1.50)	2.24 (1.71, 2.92)	1.35 (1.24, 1.46)	2.5×10^−8^
Glutamate	Glutamate metabolism	1.00	1.36 (1.01, 1.83)	1.38 (1.03, 1.84)	2.31 (1.77, 3.02)	1.36 (1.26, 1.47)	6.7×10^−10^
5-Oxoproline	Glutathione metabolism	1.00	0.88 (0.65, 1.18)	1.08 (0.81, 1.43)	1.67 (1.30, 2.16)	1.30 (1.22, 1.40)	2.3×10^−6^
Cysteine-glutathione disulfide	Glutathione metabolism	1.00	0.59 (0.46, 0.76)	0.70 (0.56, 0.89)	0.53 (0.40, 0.69)	0.84 (0.78, 0.91)	1.4×10^−5^
Cysteinylglycine	Glutathione metabolism	1.00	0.62 (0.49, 0.80)	0.64 (0.49, 0.82)	0.59 (0.46, 0.76)	0.78 (0.72, 0.84)	3.8×10^−5^
Cys-gly, oxidized	Glutathione metabolism	1.00	0.53 (0.41, 0.68)	0.61 (0.48, 0.78)	0.57 (0.44, 0.73)	0.77 (0.72, 0.83)	4.1×10^−5^
Serine	Glycine, serine, and threonine metabolism	1.00	1.10 (0.82, 1.47)	1.32 (0.99, 1.77)	1.95 (1.49, 2.54)	1.34 (1.23, 1.47)	3.7×10^−8^
Glycine	Glycine, serine and threonine metabolism	1.00	1.07 (0.80, 1.43)	1.12 (0.84, 1.48)	1.81 (1.39, 2.36)	1.26 (1.15, 1.38)	3.3×10^−6^
Histidine	Histidine Metabolism	1.00	0.97 (0.73, 1.29)	1.28 (0.98, 1.68)	1.63 (1.26, 2.12)	1.21 (1.11, 1.33)	1.4×10^−5^
Cysteine sulfinic acid	Methionine, cysteine, SAM, and taurine metabolism	1.00	1.10 (0.82, 1.46)	1.17 (0.88, 1.54)	2.06 (1.59, 2.67)	1.28 (1.19, 1.38)	2.8×10^−8^
Methionine sulfoxide	Methionine, cysteine, SAM, and taurine metabolism	1.00	1.19 (0.89, 1.59)	1.25 (0.94, 1.67)	1.96 (1.50, 2.57)	1.28 (1.18, 1.39)	2.5×10^−7^
Phenylalanine	Phenylalanine metabolism	1.00	1.02 (0.77, 1.36)	1.04 (0.78, 1.38)	1.91 (1.48, 2.47)	1.34 (1.23, 1.46)	1.1×10^−7^
N-Formylphenylalanine	Tyrosine metabolism	1.00	1.26 (0.94, 1.68)	1.35 (1.03, 1.77)	1.72 (1.32, 2.23)	1.25 (1.13, 1.38)	4.3×10^−5^
Arginine	Urea cycle; arginine and proline metabolism	1.00	1.31 (0.99, 1.75)	1.23 (0.92, 1.64)	1.99 (1.53, 2.57)	1.31 (1.20, 1.43)	4.0×10^−7^
**Carbohydrates**							
Erythronate	Amino sugar metabolism	1.00	1.40 (1.06, 1.86)	1.34 (1.01, 1.76)	1.96 (1.50, 2.57)	1.24 (1.13, 1.36)	4.4×10^−6^
**Cofactors and vitamins**							
Oxalate (ethanedioate)	Ascorbate and aldarate metabolism	1.00	0.60 (0.47, 0.77)	0.54 (0.42, 0.69)	0.58 (0.45, 0.75)	0.75 (0.69, 0.82)	3.0×10^−6^
Threonate	Ascorbate and aldarate metabolism	1.00	0.86 (0.68, 1.09)	0.62 (0.48, 0.80)	0.63 (0.49, 0.82)	0.80 (0.73, 0.87)	3.2×10^−5^
**Lipids**							
Linoleoyl ethanolamide	Endocannabinoids	1.00	0.90 (0.68, 1.21)	1.03 (0.79, 1.35)	1.62 (1.27, 2.07)	1.25 (1.14, 1.37)	3.1×10^−5^
N-Oleoylserine	Endocannabinoids	1.00	1.20 (0.90, 1.61)	1.39 (1.06, 1.84)	1.69 (1.30, 2.20)	1.14 (1.03, 1.26)	4.0×10^−5^
N-Stearoylserine	Endocannabinoids	1.00	1.16 (0.87, 1.54)	1.40 (1.06, 1.85)	1.65 (1.27, 2.14)	1.21 (1.10, 1.33)	4.6×10^−5^
Heptenedioate (C7:1-DC)	Fatty acid, dicarboxylate	1.00	1.10 (0.83, 1.45)	1.47 (1.12, 1.93)	1.71 (1.30, 2.25)	1.28 (1.15, 1.43)	9.6×10^−6^
13-HODE + 9-HODE	Fatty acid, monohydroxy	1.00	1.07 (0.81, 1.43)	1.34 (1.02, 1.76)	1.70 (1.31, 2.20)	1.28 (1.18, 1.38)	1.1×10^−5^
Glycerol 3-phosphate	Glycerolipid metabolism	1.00	1.22 (0.91, 1.63)	1.35 (1.02, 1.78)	1.74 (1.33, 2.27)	1.22 (1.12, 1.32)	3.4×10^−5^
Arachidonate (20:4n6)	Long-chain polyunsaturated fatty acid (n3 and n6)	1.00	1.14 (0.84, 1.54)	1.35 (1.02, 1.80)	2.12 (1.63, 2.77)	1.29 (1.19, 1.40)	1.7×10^−9^
Dihomolinolenate (20:3n3 or 3n6)	Long-chain polyunsaturated fatty acid (n3 and n6)	1.00	1.39 (1.05, 1.84)	1.44 (1.09, 1.92)	1.79 (1.38, 2.32)	1.23 (1.14, 1.34)	1.8×10^−5^
1-Palmitoyl-GPA (16:0)	Lysophospholipids	1.00	1.07 (0.80, 1.43)	1.19 (0.90, 1.58)	1.76 (1.37, 2.28)	1.34 (1.23, 1.46)	2.7×10^−6^
1-Arachidonoyl-GPA (20:4)	Lysophospholipids	1.00	0.81 (0.60, 1.08)	0.75 (0.56, 1.00)	1.61 (1.26, 2.06)	1.25 (1.15, 1.36)	2.7×10^−5^
Choline	Phospholipid metabolism	1.00	0.92 (0.68, 1.23)	1.32 (0.99, 1.75)	2.07 (1.60, 2.69)	1.34 (1.24, 1.43)	7.2×10^−11^
Sphinganine	Sphingolipid synthesis	1.00	1.04 (0.77, 1.39)	1.35 (1.02, 1.78)	1.63 (1.26, 2.12)	1.24 (1.15, 1.35)	1.9×10^−5^
**Nucleotides**							
N6-Methyladenosine	Purine metabolism, adenine containing	1.00	1.00 (0.73, 1.37)	1.30 (1.01, 1.68)	1.64 (1.29, 2.08)	1.30 (1.19, 1.43)	1.4×10^−5^
**Peptides**							
Glycylvaline	Dipeptide	1.00	1.21 (0.90, 1.63)	1.18 (0.88, 1.59)	1.97 (1.50, 2.58)	1.32 (1.22, 1.44)	3.0×10^−7^
Valylleucine	Dipeptide	1.00	1.11 (0.84, 1.47)	1.08 (0.81, 1.45)	1.83 (1.42, 2.37)	1.28 (1.18, 1.39)	1.8×10^−6^
Leucylglycine	Dipeptide	1.00	1.27 (0.95, 1.70)	1.21 (0.90, 1.62)	1.85 (1.42, 2.42)	1.31 (1.21, 1.42)	4.9×10^−6^
ADPSGEGDFXAEGGGVR	Fibrinogen cleavage peptide	1.00	0.60 (0.46, 0.77)	0.65 (0.51, 0.83)	0.54 (0.42, 0.70)	0.78 (0.72, 0.84)	5.1×10^−6^
Fibrinopeptide B (1–9)	Fibrinogen cleavage peptide	1.00	1.17 (0.87, 1.58)	1.31 (0.98, 1.75)	1.80 (1.38, 2.36)	1.27 (1.16, 1.39)	4.0×10^−6^
Fibrinopeptide B (1–12)	Fibrinogen cleavage peptide	1.00	0.57 (0.44, 0.73)	0.59 (0.46, 0.75)	0.60 (0.47, 0.77)	0.82 (0.77, 0.87)	2.3×10^−5^
Fibrinopeptide B (1–11)	Fibrinogen cleavage peptide	1.00	0.54 (0.42, 0.69)	0.66 (0.52, 0.85)	0.57 (0.45, 0.73)	0.82 (0.77, 0.87)	5.2×10^−5^
Gamma-glutamylglutamate	Gamma-glutamyl amino acid	1.00	1.05 (0.78, 1.42)	1.24 (0.93, 1.66)	2.07 (1.60, 2.69)	1.35 (1.26, 1.45)	1.3×10^−9^
Gamma-glutamylglycine	Gamma-glutamyl amino acid	1.00	1.23 (0.91, 1.66)	1.46 (1.10, 1.95)	2.12 (1.62, 2.77)	1.32 (1.23, 1.41)	4.0×10^−9^
Gamma-glutamylleucine	Gamma-glutamyl amino acid	1.00	1.31 (0.98, 1.76)	1.17 (0.87, 1.56)	2.14 (1.65, 2.78)	1.31 (1.22, 1.40)	2.4×10^−8^
Gamma-glutamylvaline	Gamma-glutamyl amino acid	1.00	1.39 (1.04, 1.87)	1.18 (0.88, 1.58)	2.14 (1.64, 2.79)	1.33 (1.24, 1.42)	7.8×10^−8^
Gamma-glutamylphenylalanine	Gamma-glutamyl amino acid	1.00	1.35 (1.02, 1.81)	1.30 (0.96, 1.75)	2.08 (1.59, 2.71)	1.32 (1.23, 1.42)	8.7×10^−8^
Gamma-glutamylserine	Gamma-glutamyl amino acid	1.00	1.13 (0.85, 1.52)	1.05 (0.78, 1.42)	1.99 (1.53, 2.59)	1.35 (1.21, 1.49)	1.5×10^−7^
Gamma-glutamylisoleucine	Gamma-glutamyl amino acid	1.00	1.20 (0.90, 1.62)	1.09 (0.82, 1.46)	1.97 (1.51, 2.58)	1.31 (1.22, 1.41)	5.7×10^−7^
Gamma-glutamylthreonine	Gamma-glutamyl amino acid	1.00	1.15 (0.86, 1.54)	1.26 (0.94, 1.67)	1.84 (1.42, 2.39)	1.30 (1.21, 1.40)	1.2×10^−6^
Gamma-glutamylmethionine	Gamma-glutamyl amino acid	1.00	1.21 (0.91, 1.61)	1.25 (0.95, 1.66)	1.89 (1.46, 2.46)	1.28 (1.18, 1.38)	1.3×10^−6^
Gamma-glutamyl-alpha-lysine	Gamma-glutamyl amino acid	1.00	0.74 (0.55, 0.99)	0.92 (0.69, 1.21)	1.58 (1.24, 2.03)	1.29 (1.20, 1.39)	8.0×10^−6^
Gamma-glutamyltyrosine	Gamma-glutamyl amino acid	1.00	1.22 (0.92, 1.61)	1.13 (0.84, 1.52)	1.74 (1.35, 2.25)	1.29 (1.20, 1.40)	3.1×10^−5^
**Xenobiotics**							
S-Carboxymethyl-L-cysteine	Drug - other	1.00	0.57 (0.45, 0.73)	0.63 (0.49, 0.82)	0.53 (0.41, 0.69)	0.81 (0.75, 0.88)	3.4×10^−6^

*Abbreviations*: *ATBC* Alpha-Tocopherol, Beta-Carotene Cancer Prevention, *HR* hazard ratio, *SD* standard deviation, *SAM* S-adenosylmethionine

^a^ HRs and 95% CIs were estimated from Cox proportional hazards regression models adjusted for age at baseline, age at diagnosis, cancer stage at diagnosis (stage I–IV), and Gleason scores at cancer diagnosis. Metabolites are shown sorted by chemical class and sub-class pathway and then by *P*-value

^b^ *P*-value for trend was calculated by including in the regression model the ordinal value of the quartile of each metabolite and treating this as a continuous variable

Further model adjustment for BMI, number of cigarettes smoked daily, years of smoking, serum total cholesterol, serum high-density lipoprotein cholesterol and calendar year of prostate cancer diagnosis did not alter the observed associations (Additional File 1: Tables S1).

**Fig. 1:**
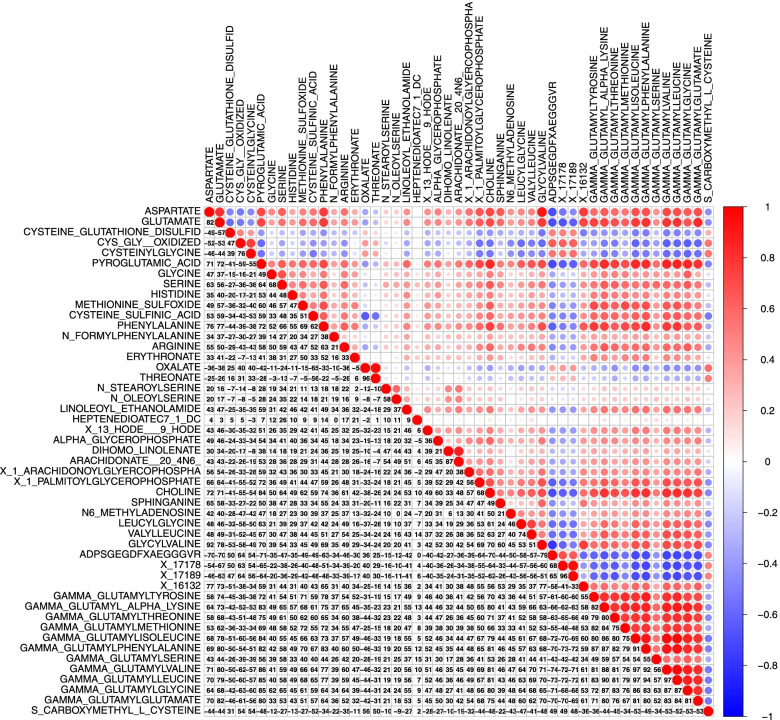
Heatmap of Pearson correlation coefficients for top metabolites associated with prostate cancer-specific mortality that achieved Bonferroni corrected threshold. Directions and magnitudes of the correlation coefficients are represented by the color (i.e., red=positive correlations; blue=negative correlations) and circle sizes (i.e., larger circles for stronger correlations)

Figure 1 shows the heat-map of Pearson correlation coefficients for metabolites that were significantly associated with prostate cancer-specific mortality after Bonferroni correction. Stronger positive correlations were found within the gamma-glutamyl amino acid chemical sub-class pathway.

Stratifying cases by cancer stage at diagnosis, the observed metabolite associations with prostate cancer mortality were generally stronger for men with earlier stage cancers (i.e., I/II), with statistically significant stronger associations for 5-oxoproline (1-SD HR 1.54 and 1.17 for earlier versus late cancer stage), fibrinopeptide B (1–12) (HR 0.70 and 0.90), fibrinopeptide B (1–11) (HR 0.70 and 0.91), gamma-glutamylisoleucine (HR 1.57 and 1.17), and gamma-glutamylleucine (HR 1.55 and 1.18) (all *P* for interaction <3.4×10^−4^, Bonferroni correction for 49×3 tests, Additional File 1: Tables S2). There was no statistically significant effect modification of metabolite associations by BMI or time from blood collection to cancer diagnosis (Additional File 1: Tables S1 ).

**Fig. 2:**
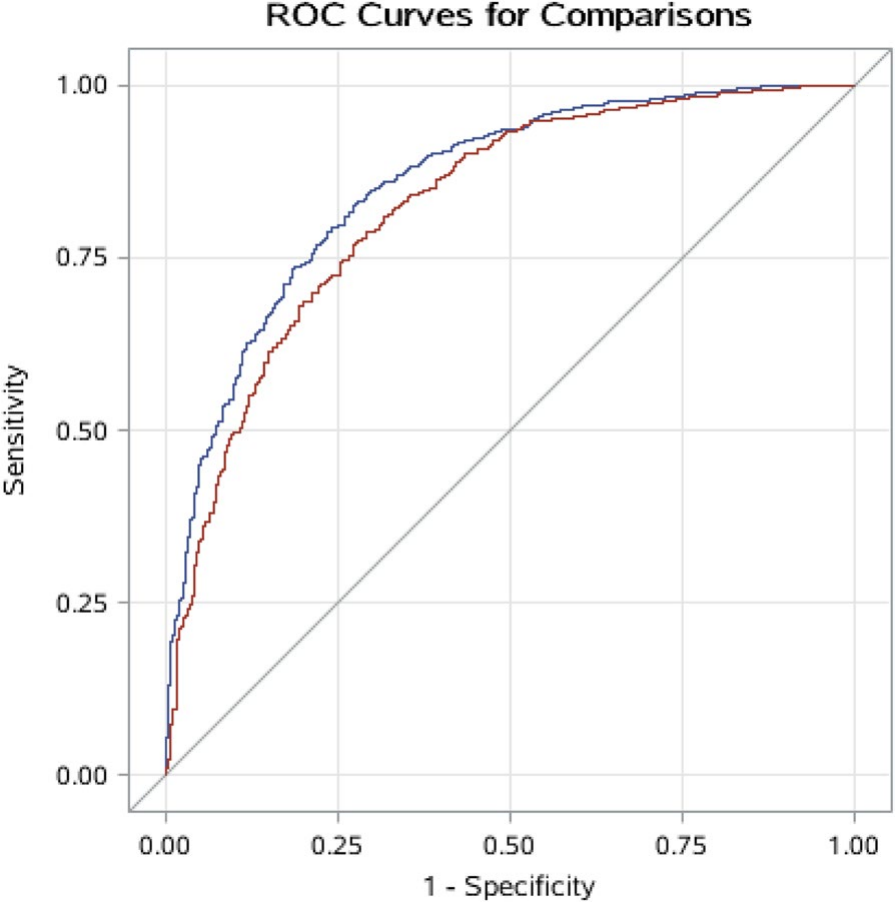
Adjusted receiver operating characteristics (ROC) and adjusted area under the curve (AUC) for clinical factors and selected metabolites associated with risk of prostate cancer-specific mortality. Results for red ROC curve: AUC=0.82 (95% CI = 0.80 to 0.85) for clinical factors including age at blood collection, age at diagnosis, cancer stage at diagnosis, Gleason score at diagnosis, cigarettes smoked per day and BMI. Results for blue ROC curve: AUC=0.86 (95% CI = 0.84 to 0.88) for clinical factors and five metabolites selected from the forward stepwise regression models, including gamma-glutamylglutamate, heptenedioate (C7:1-DC), oxalate, arachidonate (20:4n6) and linoleoyl ethanolamide (AUC 0.86 versus 0.82, *P* value = 0.04)

Using the forward stepwise selection analysis, we identified five metabolites that were independently statistically significantly associated with prostate cancer death up to model step five, including gamma-glutamylglutamate, heptenedioate (C7:1-DC), oxalate, arachidonate (20:4n6), and linoleoyl ethanolamide (Additional File 1: Tables S3). The corresponding adjusted ROC had an adjusted AUC of 0.82 (95% CI = 0.80 to 0.85) for clinical factors including age at blood collection, age at diagnosis, cancer stage at diagnosis, Gleason score at diagnosis, cigarettes smoked per day, and BMI, and 0.86 (95% CI = 0.84 to 0.88) for those clinical factors and five metabolites selected from the forward stepwise regression models, including gamma-glutamylglutamate, heptenedioate (C7:1-DC), oxalate, arachidonate (20:4n6) and linoleoyl ethanolamide. The adjusted AUC was significantly influenced and improved from 0.82 to 0.86 by the five selected metabolites (AUC 0.86 versus 0.82, *P* value = 0.04; Fig. 2).

Pathway and principal component analyses highlighted the potentially relevant importance of eight metabolic chemical pathways that were significantly associated with prostate cancer mortality at the Bonferroni-corrected threshold of 5.3×10^−4^, including endocannabinoid, glycine-serine-and-threonine, gamma-glutamyl amino acid, glutamate, eicosanoid, sphingolipid synthesis, glutathione, and dipeptide (Table 3). Seven of these pathways were positively associated with prostate cancer-specific mortality (fourth versus first quartile: HRs=1.61–1.94, 4.5×10^−7^≤*P*-value≤2.0×10^−4^), and only the glutathione metabolism pathway was inversely associated (fourth versus first quartile: HR=0.62, *P*-value=1.7×10^−4^).

**Table 3:** Pathway analysis and principal components analysis (PCA) for the association between chemical sub-classes of serum metabolites and prostate cancer-specific mortality in the ATBC Study ^a^

Chemical sub-class pathway	Pathway analysis		PCA analysis for pattern score (by quartiles) ^**b**^				
	No. of contributing metabolites	***P***-value	Q1	Q2	Q3	Q4	***P***-value ^c^
			Referent	HR (95% CI)	HR (95% CI)	HR (95% CI)	
Endocannabinoids	10	<10^−5^	1.00	1.25 (0.94, 1.67)	1.54 (1.16, 2.04)	1.92 (1.46, 2.51)	4.5×10^−7^
Glycine, serine, and threonine metabolism	10	<10^−5^	1.00	1.22 (0.92, 1.63)	1.43 (1.08, 1.88)	1.92 (1.47, 2.51)	6.5×10^−7^
Gamma-glutamyl amino acids	18	<10^−5^	1.00	1.30 (0.97, 1.72)	1.26 (0.94, 1.69)	1.94 (1.49, 2.52)	9.5×10^−7^
Glutamate metabolism	13	<10^−5^	1.00	1.14 (0.87, 1.51)	1.39 (1.05, 1.83)	1.71 (1.32, 2.23)	1.5×10^−5^
Eicosanoid	3	<10^−5^	1.00	0.93 (0.70, 1.25)	1.22 (0.93, 1.60)	1.62 (1.25, 2.10)	1.8×10^−5^
Sphingolipid synthesis	3	<10^−5^	1.00	1.08 (0.81, 1.44)	1.36 (1.03, 1.78)	1.61 (1.24, 2.08)	5.5×10^−5^
Glutathione metabolism	7	<10^−5^	1.00	0.59 (0.46, 0.75)	0.63 (0.49, 0.80)	0.62 (0.48, 0.79)	1.7×10^−4^
Dipeptides	6	<10^−5^	1.00	1.17 (0.87, 1.56)	1.08 (0.81, 1.45)	1.65 (1.27, 2.15)	2.0×10^−4^

*Abbreviations*: *HR* hazard ratio, *CI* confidence interval, *ATBC* Alpha-Tocopherol, Beta-Carotene Cancer Prevention

^a^ Pathway analysis: We examined the association between 95 chemical sub-classes of serum metabolites and prostate cancer-specific mortality and tested a single *P*-value for pathways using a parametric bootstrap method. Within each bootstrap replication, *P*-values were generated from a vector of score test statistics from an estimated covariance matrix with a multivariate normal distribution (mean=0). The pathway *P*-values are calculated based on 100,000 permutations

^b^ Models were adjusted for age at baseline, age at diagnosis, cancer stage at diagnosis (stage I–IV), and Gleason scores at cancer diagnosis

^c^ Bonferroni-corrected threshold = 5.3×10^−4^, 0.05/95

We combined the five metabolites selected from Cox regression model in the 70% discovery set with those achieving Bonferroni corrected threshold (<5.2×10^−5^) to generate a metabolite risk score and observed statistically significantly increased mortality in the replication set for cases having a higher score (per 1-SD HR=1.48, 95% CI: 1.30 to 1.68, Table 4). In the categorized analysis of the risk score quartiles, we found cases in the second through fourth quartiles experienced an 83%, 85%, and 290% higher risk of prostate cancer-specific mortality, when compared with those in the lowest quartile (*P* for trend<0.0001, Table 4).

**Table 4:** Association between metabolite risk score and prostate cancer-specific mortality in the 30% replication set of 543 prostate cancer cases

Quartile of metabolite risk score ^**a**^	Deaths	Total No. of cases	Person-years	HRs ^b^	95% CI
**1**	20	135	1056.4	1.00	Referent
**2**	33	136	1054.9	1.83	1.04, 3.25
**3**	29	136	987.2	1.85	1.03, 3.33
**4**	60	136	918.4	3.90	2.32, 6.55
***P*** **for trend**				<0.0001	
**Metabolite risk score as continuous variable (per 1-SD)**				1.48	1.30, 1.68
***P*-value (per 1-SD)**				<0.0001	

*Abbreviations*: *HR* hazard ratio, *CI* confidence interval, *SD* standard deviation

^a^ The metabolite risk score was constructed in the replication set by summing the top metabolites that achieved Bonferroni corrected threshold (0.05/961) weighted by their Cox regression coefficients from the discovery set. The metabolic risk score is generated as

(Log-glutamate)× 0.256 + (Log-gamma-glutamylglutamate)× (0.237) + (Log-methionine sulfoxide)× (0.236) + (Log-arachidonate (20:4n6)×0.227) + (Log-choline)×(0.223)

^b^ Hazard ratio for prostate cancer-specific mortality using Cox proportional hazards regression model and adjusted for age at baseline, age at diagnosis, cancer stage at diagnosis (stage I–IV), and Gleason scores at cancer diagnosis

## Discussion

In this large prospective metabolomic analysis, we found 49 metabolites that were statistically significantly associated with prostate cancer mortality, with top signals including choline, glutamate, arachidonate, and gamma-glutamyl amino acids. Additionally, we identified metabolites in the endocannabinoid, glycine-serine-and-threonine-metabolism, eicosanoid, sphingolipid synthesis, dipeptide, and redox pathways being most strongly associated with risk of prostate cancer mortality. Of note, the risk in men with prostate cancer was elevated fourfold in those in the highest metabolite risk score category.

As the top signal in this present metabolomic analysis, serum choline was positively associated with risk of prostate cancer mortality. Abnormal choline metabolism has been characterized as a cholinic phenotype and a metabolic hallmark, related to increased oncogenic signaling and tumor progression [22]. In a case-only survival analysis within the Health Professionals Follow-Up Study, the authors showed that post-diagnostic intake of dietary choline was related to an increased risk of lethal prostate cancer, with a 70% increased risk for men in the highest quintile of intake [23]. Furthermore, a prospective nested case-control study within the ATBC cohort showed that men with greater serum choline levels had a 3-fold increased risk of developing colorectal cancer [24].

The second top signal identified was glutamate, with higher levels related to an elevated risk of prostate cancer mortality. Glutamate is catabolized from glutamine and in turn incorporated into the tricarboxylic acid (TCA) cycle and lipogenesis [25]. A large portion of glutamine-derived glutamate is secreted from cells via cystine/glutamate transporters in exchange for cystine, which plays vital roles in redox homeostasis [26]. Moreover, the secreted glutamate can stimulate cancer cell proliferation via glutamate receptors [26], consistent with serum glutamate being correlated with Gleason sum in prostate cancer patients [25] and in vivo studies demonstrating that reduced glutamate excretion inhibited tumor xenograft growth, including prostate cancer [27, 28]. In addition, expression of the cystine/glutamate transporter SLC7A11 is upregulated in different cancers including prostate [26, 29].

Consistent with our previous analysis of fatal prostate cancer risk in a smaller set of 523 cases-control sets [13], serum dipeptides including leucylglycine, valylleucine, glycylvaline, and gamma-glutamyl amino acids were associated with an increased risk of prostate cancer-specific mortality here. As a key enzyme in the gamma-glutamyl cycle, gamma-glutamyl peptidase (GGT) is known for degradation of extracellular glutathione and liberation of free gamma-glutamyl peptides and is characterized as a clinical indicator of prostate cancer [13]. Experimental data also demonstrate that GGT can be involved in cellular detoxification and apoptotic balance pathways [30]. For example, in vitro and in vivo studies report that inhibition of the GGT pathway can impact cancer cell growth through cell-cycle regulation, and enhance the sensitivity to chemotherapy [31]. Consistent with our findings, earlier human data showed that circulating gamma-glutamyl peptides were associated with an increased risk of hepatocellular carcinoma, and elevated serum GGT was related to an increased risk of overall and site-specific cancers, including prostate cancer [32].

Perturbations in endocannabinoid and glycine-serine-threonine metabolism have been related to the risk of prostate cancer survival. Endocannabinoids are endogenous ligands of cannabinoid receptors (CB1 and CB2) [33], and dysregulation of the endocannabinoid system has been associated with multiple health outcomes including cardiovascular disease, neurodegenerative disorders, and cancer [33]. Several components of the endocannabinoid system were overexpressed in prostate cancer cell lines and correlated with prostate cancer grade and progression in epithelial cells of prostate cancer tissue samples [34]. Our current and previous findings show positive associations between serum endocannabinoids (i.e., N-oleoyl taurine, N-stearoyl serine, N-oleoyl serine, and linoleoyl ethanolamide) and risk of prostate cancer survival [17]. Furthermore, biosynthesis and metabolism of serine, aspartate, and glycine are required for cancer cell growth and progression [35, 36], for example, malignant cells upregulate de novo serine and glycine synthesis and secretion [37, 38] which may help maintain a supportive tumor microenvironment [37].

The positive association we observed between eicosanoids and prostate cancer mortality is also biologically plausible in that eicosanoids have been found to promote cancer cell growth in an autocrine fashion by activating their receptors implicated in the regulation of cell proliferation, apoptosis, migration, and invasion [39]. Also, eicosanoid signaling inhibitors (e.g., CAY10404 and celecoxib) decrease prostate cancer cell proliferation [40], and eicosanoids are reported to have immunosuppressive effects which alter the tumor microenvironment and T cell profiles, potentially serving as a therapeutic target for prostate cancer [39].

Alterations in sphingolipid biosynthesis and metabolism have been associated with elevated prostate cancer mortality, and serum sphingolipids concentrations are related to colorectal cancer and hepatocellular carcinoma [41, 42]. Sphingolipids with their fatty acid tail and sphingoid ring base have significant cell membrane roles in regulating various cellular biological processes including cell proliferation, apoptosis, and inflammatory response [43, 44], and emerging evidence supports their potential use as cancer therapeutic targets [44]. The essential amino acid methionine also plays a critical role in tumor cell proliferation and metabolism [45], including glutathione formation, polyamine synthesis, and methyl group donation [45]. Reduced methionine status through dietary restriction or enzymatic degradation can inhibit prostate tumor growth [46]. Another of our metabolite signals, arginine, also plays multiple roles in cellular physiology, including nitric oxide and polyamines synthesis, cell proliferation, cellular signaling, and immune system regulation [47, 48], and can activate rapamycin complex 1 (mTORC1), a nutrient-sensing kinase directly involved in tumorigenesis [48].

We also observed associations between elevated fatty acid metabolites arachidonate, 9-hydroxyoctadecadienoic acid (HODE), and 13-HODE and increased prostate cancer mortality. 9-HODE and 13-HODE can be synthesized by cyclooxygenase (COX) and 15-lipoxygenase 1(15-LOX 1) from linoleic acid [49, 50], and activation of the 15-lipoxygenase 1/13(S)-HODE axis can promote prostate cancer cell growth through the epidermal growth factor receptor (EGFR) signaling pathway [50]. Consistent with our findings, a nested case-control study in the Prostate, Lung, Colorectal, and Ovarian (PLCO) Cancer Screening Trial cohort reported positive associations between serum concentrations of 9-HODE and 13-HODE and increased risk of developing ovarian cancer, highlighting the possible roles of linoleic acid metabolites in cancer etiology [51]. In addition, N6-methyladenosine (m6A) is one of the most prevalent internal posttranscriptional modifications of RNA and participates in RNA metabolism, including mRNA translation, splicing, folding, and degradation [52]. Growing evidence suggests a key role for m6A in tumorigenesis, including for prostate cancer [52] and its progression [53], and tumor data from the Cancer Genome Atlas database indicate overexpression of m6A methylation regulators in the aggressive prostate tumor tissues [54]. These findings are in line with the current data suggesting a positive association between serum m6A and prostate cancer mortality.

We have validated findings from our previous metabolomics studies including gamma-glutamyl amino acids, fibrinopeptides, endocannabinoids, and redox metabolites. Additionally, based on the larger sample size of the present study, we identified new metabolites associated with poor prostate cancer survival, including choline, glutamate, and arachidonate. When we compared the present findings with the 34 metabolites identified in our previous study of lethal prostate cancer in 523 case-control sets [13], 25 metabolites were validated in the present study, three metabolites were not measured in the current panel (histidylalanine, valyglycine, and leucylglutamine), four metabolites were marginally associated in the same direction but with lower statistical significance (*P* value range of 0.08 to 0.11; C-glycosyltryptophan, 1-linoleoyl-GPC (18:2), 5,6-dihydrouridine and 5-methyluridine), and only two metabolites were not validated by the present study (cystine and pseudouridine, HRs=1). Differences in our findings between the current analysis and our previous studies may be due to its larger sample size and different study design; e.g., our previous study compared lethal prostate cancer cases to cancer-free controls, whereas the present study examined prostate cancer survival among men diagnosed with the disease. Further studies will be needed to understand these nuances.

Our study has several important and novel strengths, including its quite large sample size, a prospective study design, and complete, long-term follow-up identification of prostate cancer diagnoses and mortality outcomes. We used a stringent statistical threshold, namely the Bonferroni correction, to reduce the potential for false-positive findings. Importantly, metabolomic profiles were measured in serum samples that were collected up to a median of 14 years (interquartile range = 10 to 17 years) prior to prostate cancer diagnosis, thus minimizing the possibility of reverse causality. In addition, the untargeted metabolic platform with excellent laboratory reproducibility was highly reliable and enabled us to measure more than 900 known metabolites simultaneously reflecting a wide range of biological pathways relevant to prostate carcinogenesis. Our study is one of the few prospective cohort studies that collected blood samples from participants after an overnight fast, minimizing post-prandial fluctuations in the metabolome which affect most other studies. It should also be noted that the ATBC Study was conducted in Finland, where population-based PSA screening was not implemented, resulting in most of the cases being clinically diagnosed which allowed us to examine which metabolites were related to fatal disease compared to other clinically relevant cases. In most US cohorts, by contrast, a great deal of investigational “noise” is introduced through the inclusion of indolent, PSA-detected cases. As most of the studies have been focused on the metabolomics and prostate cancer incidence, our findings highlight multiple potentially important novel metabolites and biological pathways that may play key roles in the progression and mortality of prostate cancer. Some study limitations should also be mentioned. We did not have an external validation set and relied on the internal replication set; to our knowledge, however, other studies have not measured this large a number of metabolites using an HRAM LC-MS/MS platform. Prostate cancer molecular sub-type data were not available for examination of unique metabolite relationships (i.e., ETS-related gene [ERG]-positive or negative tumors). We cannot preclude residual confounding from other factors that may have influenced the observed associations, although we have controlled for several potential confounding factors in our models, and found no strong evidence of confounding. The homogenous nature of the study population of male Finnish smokers aged 50–69 years may limit the generalizability of our findings to non-smokers, younger men, and other racial/ethnic groups.

## Conclusions

Our prospective serum metabolomic investigation of 1812 men with prostate cancer elucidated a panel of metabolites and a composite metabolite risk score from blood collected prior to diagnosis that were associated with later risk of prostate cancer-specific mortality. These findings provide novel, biologically based insights and potential leads regarding the molecular basis of prostate cancer progression and mortality, and may identify a high-risk group of men in whom prevention strategies may be implemented and for whom more specific screening recommendations may be developed.

## Supplementary Information

**Additional file 1: Table S1.** [Hazard Ratios and 95% Confidence Intervals for the Association Between Prostate Cancer Mortality and Prediagnostic Serum Metabolites Reaching the Bonferroni Corrected Threshold Based on 1,812 Prostate Cancer Cases in the ATBC Study, with further adjustment in the models]. **Table S2.** [Hazard Ratios and 95% Confidence Intervals for the Association Between Prostate Cancer Mortality and Prediagnostic Serum Metabolites Reaching the Bonferroni Corrected Threshold Stratified by Selected Factors in the ATBC Study]. **Table S3.** [Hazard Ratios and 95% Confidence Intervals for Serum Metabolites Achieving the Bonferroni Corrected Threshold Using Stepwise Cox Proportional Hazards Regression Analysis in the ATBC Study].

## Data Availability

Because of previously enacted EU General Data Protection Regulation privacy rules and an existing data use agreement between Finland and the US National Cancer Institute, the ATBC Study data and materials described in the article may not be made publicly available for the purpose of reproducing the findings. The principal investigators of the ATBC Study can be contacted with specific data requests (https://atbcstudy.cancer.gov/). To minimize the possibility of unintentionally sharing information that can be used to identify private information, a subset of the data generated for this study will be made available first on reasonable request.

## Abbreviations

ATBC: Alpha-Tocopherol, Beta-Carotene Cancer Prevention Study
AUC: Area under the curve
BMI: Body mass index
CI: Confidence interval
COX: Cyclooxygenase
CV: Coefficient of variation
EGFR: Epidermal growth factor receptor
ERG: ETS-related gene
GGT: Gamma-glutamyl peptidase
HODE: Hydroxyoctadecadienoic acid
HPLC: High-performance liquid chromatography
HR: Hazard ratio
HRAM: High-resolution accurate mass
ICC: Intraclass correlation coefficient
ICD: International Classification of Disease
KEGG: Kyoto Encyclopedia of Genes and Genomics
LC-MS: Liquid chromatography/tandem mass spectrometry
LOX: Lipoxygenase
PCA: Principal component analysis
PLCO: Prostate, Lung, Colorectal, and Ovarian Cancer Screening Trial cohort
PSA: Prostate-specific antigen
ROC: Receiver operating characteristics
ROS: Reactive oxygen species
SD: Standard deviation
TCA: Tricarboxylic acid
