# Supplementary material for: Metabolomic profile of prostate cancer-specific survival among 1812 Finnish men
Source: BMC Med. 2022 Oct 25;20:362. doi: 10.1186/s12916-022-02561-4 (PMC9594924; doi:10.1186/s12916-022-02561-4)
Supplement: Supplementary file 1 — Additional file 1: Table S1. [Hazard Ratios and 95% Confidence Intervals for the Association Between Prostate Cancer Mortality and Prediagnostic Serum Metabolites Reaching the Bonferroni Corrected Threshold Based on 1,812 Prostate Cancer Cases in the ATBC Study, with further adjustment in the models]. Table S2. [Hazard Ratios and 95% Confidence Intervals for the Association Between Prostate Cancer Mortality and Prediagnostic Serum Metabolites Reaching the Bonferroni Corrected Threshold Stratified by Selected Factors in the ATBC Study]. Table S3. [Hazard Ratios and 95% Confidence Intervals for Serum Metabolites Achieving the Bonferroni Corrected Threshold Using Stepwise Cox Proportional Hazards Regression Analysis in the ATBC Study]. [file 12916_2022_2561_MOESM1_ESM.docx]

**Table S1.** Hazard Ratios and 95% Confidence Intervals for the Association Between Prostate Cancer Mortality and Prediagnostic Serum Metabolites Reaching the Bonferroni Corrected Threshold Based on 1,812 Prostate Cancer Cases in the ATBC Study (further adjustment in the models) **^a, b^**

| **Metabolite** | **Sub-class pathway** | **Quartile of Metabolite** | | | | **1-SD** | **P value for trend** **^c^** |
| --- | --- | --- | --- | --- | --- | --- | --- |
|  |  | **Quartile 1** | **Quartile 2** | **Quartile 3** | **Quartile 4** |  |  |
| **Amino acids and amino acid derivatives** |  | HR | HR (95% CI) | HR (95% CI) | HR (95% CI) | HR (95% CI) |  |
| Aspartate: Model 1 | Alanine and Aspartate Metabolism | 1.00 | 1.44 (1.07, 1.94) | 1.12 (0.83, 1.50) | 2.24 (1.71, 2.92) | 1.35 (1.24, 1.46) | 2.5×10^-8^ |
| Model 2 |  | 1.00 | **1.42 (1.06, 1.92)** | **1.09 (0.80, 1.47)** | **2.16 (1.65, 2.84)** | **1.34 (1.23, 1.46)** | **1.9×10^-7^** |
| Glutamate: Model 1 | Glutamate Metabolism | 1.00 | 1.36 (1.01, 1.83) | 1.38 (1.03, 1.84) | 2.31 (1.77, 3.02) | 1.36 (1.26, 1.47) | 6.7×10^-10^ |
| Model 2 |  | 1.00 | **1.35 (1.00, 1.83)** | **1.38 (1.03, 1.86)** | **2.32 (1.76, 3.07)** | **1.37 (1.26, 1.48)** | **1.5×10^-9^** |
| Cysteine-Glutathione Disulfide: Model 1 | Glutathione Metabolism | 1.00 | 0.59 (0.46, 0.76) | 0.70 (0.56, 0.89) | 0.53 (0.40, 0.69) | 0.84 (0.78, 0.91) | 1.4×10^-5^ |
| Model 2 |  | 1.00 | **0.58 (0.45, 0.75)** | **0.71 (0.55, 0.90)** | **0.53 (0.40, 0.70)** | **0.84 (0.78, 0.91)** | **2.8×10^-5^** |
| Cys-Gly, Oxidized: Model 1 | Glutathione Metabolism | 1.00 | 0.53 (0.41, 0.68) | 0.61 (0.48, 0.78) | 0.57 (0.44, 0.73) | 0.77 (0.72, 0.83) | 4.1×10^-5^ |
| Model 2 |  | 1.00 | **0.52 (0.41, 0.68)** | **0.61 (0.48, 0.78)** | **0.57 (0.44, 0.73)** | **0.77 (0.72, 0.84)** | **5.0×10^-5^** |
| Cysteinylglycine: Model 1 | Glutathione Metabolism | 1.00 | 0.62 (0.49, 0.80) | 0.64 (0.49, 0.82) | 0.59 (0.46, 0.76) | 0.78 (0.72, 0.84) | 3.8×10^-5^ |
| Model 2 |  | 1.00 | **0.62 (0.49, 0.80)** | **0.64 (0.49, 0.82)** | **0.58 (0.45, 0.75)** | **0.78 (0.72, 0.84)** | **3.4×10^-5^** |
| 5-Oxoproline: Model 1 | Glutathione Metabolism | 1.00 | 0.88 (0.65, 1.18) | 1.08 (0.81, 1.43) | 1.67 (1.30, 2.16) | 1.30 (1.22, 1.40) | 2.3×10^-6^ |
| Model 2 |  | 1.00 | **0.88 (0.65, 1.18)** | **1.08 (0.81, 1.43)** | **1.64 (1.27, 2.12)** | **1.30 (1.21, 1.40)** | **6.4×10^-6^** |
| Glycine: Model 1 | Glycine, Serine and Threonine Metabolism | 1.00 | 1.07 (0.80, 1.43) | 1.12 (0.84, 1.48) | 1.81 (1.39, 2.36) | 1.26 (1.15, 1.38) | 3.3×10^-6^ |
| Model 2 |  | 1.00 | **1.07 (0.80, 1.43)** | **1.15 (0.86, 1.53)** | **1.87 (1.42, 2.45)** | **1.27 (1.16, 1.40)** | **1.4×10^-6^** |
| Serine: Model 1 | Glycine, Serine and Threonine Metabolism | 1.00 | 1.10 (0.82, 1.47) | 1.32 (0.99, 1.77) | 1.95 (1.49, 2.54) | 1.34 (1.23, 1.47) | 3.7×10^-8^ |
| Model 2 |  | 1.00 | **1.10 (0.82, 1.48)** | **1.36 (1.01, 1.82)** | **1.95 (1.49, 2.55)** | **1.34 (1.23, 1.46)** | **4.3×10^-8^** |
| Histidine: Model 1 | Histidine Metabolism | 1.00 | 0.97 (0.73, 1.29) | 1.28 (0.98, 1.68) | 1.63 (1.26, 2.12) | 1.21 (1.11, 1.33) | 1.4×10^-5^ |
| Model 2 |  | 1.00 | **0.97 (0.73, 1.29)** | **1.26 (0.96, 1.65)** | **1.60 (1.23, 2.08)** | **1.20 (1.10, 1.32)** | **4.2×10^-5^** |
| Methionine Sulfoxide: Model 1 | Methionine, Cysteine, SAM and Taurine Metabolism | 1.00 | 1.19 (0.89, 1.59) | 1.25 (0.94, 1.67) | 1.96 (1.50, 2.57) | 1.28 (1.18, 1.39) | 2.5×10^-7^ |
| Model 2 |  | 1.00 | **1.18 (0.88, 1.59)** | **1.26 (0.94, 1.69)** | **2.00 (1.52, 2.62)** | **1.29 (1.19, 1.41)** | **1.4×10^-7^** |
| Cysteine Sulfinic Acid: Model 1 | Methionine, Cysteine, SAM and Taurine Metabolism | 1.00 | 1.10 (0.82, 1.46) | 1.17 (0.88, 1.54) | 2.06 (1.59, 2.67) | 1.28 (1.19, 1.38) | 2.8×10^-8^ |
| Model 2 |  | 1.00 | **1.10 (0.83, 1.47)** | **1.18 (0.89, 1.56)** | **2.06 (1.59, 2.68)** | **1.28 (1.19, 1.38)** | **3.7×10^-8^** |
| Phenylalanine: Model 1 | Phenylalanine Metabolism | 1.00 | 1.02 (0.77, 1.36) | 1.04 (0.78, 1.38) | 1.91 (1.48, 2.47) | 1.34 (1.23, 1.46) | 1.1×10^-7^ |
| Model 2 |  | 1.00 | **1.00 (0.75, 1.34)** | **1.01 (0.76, 1.35)** | **1.87 (1.44, 2.43)** | **1.33 (1.22, 1.45)** | **4.7×10^-7^** |
| N-Formylphenylalanine: Model 1 | Tyrosine Metabolism | 1.00 | 1.26 (0.94, 1.68) | 1.35 (1.03, 1.77) | 1.72 (1.32, 2.23) | 1.25 (1.13, 1.38) | 4.3×10^-5^ |
| Model 2 |  | 1.00 | **1.25 (0.93, 1.67)** | **1.34 (1.02, 1.76)** | **1.72 (1.32, 2.23)** | **1.24 (1.12, 1.37)** | **5.1×10^-5^** |
| Arginine: Model 1 | Urea cycle; Arginine and Proline Metabolism | 1.00 | 1.31 (0.99, 1.75) | 1.23 (0.92, 1.64) | 1.99 (1.53, 2.57) | 1.31 (1.20, 1.43) | 4.0×10^-7^ |
| Model 2 |  | 1.00 | **1.31 (0.98, 1.74)** | **1.22 (0.91, 1.63)** | **1.95 (1.50, 2.54)** | **1.30 (1.19, 1.42)** | **1.3×10^-6^** |
| **Carbohydrates** |  |  |  |  |  |  |  |
| Erythronate: Model 1 | Amino Sugar Metabolism | 1.00 | 1.40 (1.06, 1.86) | 1.34 (1.01, 1.76) | 1.96 (1.50, 2.57) | 1.24 (1.13, 1.36) | 4.4×10^-6^ |
| Model 2 |  | 1.00 | **1.40 (1.05, 1.86)** | **1.33 (1.01, 1.76)** | **1.95 (1.48, 2.57)** | **1.24 (1.12, 1.36)** | **8.5×10^-6^** |
| **Cofactors and Vitamins** |  |  |  |  |  |  |  |
| Oxalate (ethanedioate): Model 1 | Ascorbate and Aldarate Metabolism | 1.00 | 0.60 (0.47, 0.77) | 0.54 (0.42, 0.69) | 0.58 (0.45, 0.75) | 0.75 (0.69, 0.82) | 3.0×10^-6^ |
| Model 2 |  | 1.00 | **0.60 (0.47, 0.77)** | **0.54 (0.42, 0.69)** | **0.57 (0.44, 0.74)** | **0.75 (0.68, 0.82)** | **1.8×10^-6^** |
| Threonate: Model 1 | Ascorbate and Aldarate Metabolism | 1.00 | 0.86 (0.68, 1.09) | 0.62 (0.48, 0.80) | 0.63 (0.49, 0.82) | 0.80 (0.73, 0.87) | 3.2×10^-5^ |
| Model 2 |  | 1.00 | **0.86 (0.68, 1.10)** | **0.62 (0.48, 0.80)** | **0.62 (0.48, 0.81)** | **0.79 (0.73, 0.87)** | **2.1×10^-5^** |
| **Lipids** |  |  |  |  |  |  |  |
| N-Stearoylserine: Model 1 | Endocannabinoid | 1.00 | 1.16 (0.87, 1.54) | 1.40 (1.06, 1.85) | 1.65 (1.27, 2.14) | 1.21 (1.10, 1.33) | 4.6×10^-5^ |
| Model 2 |  | 1.00 | **1.18 (0.88, 1.57)** | **1.42 (1.08, 1.88)** | **1.68 (1.28, 2.20)** | **1.22 (1.10, 1.34)** | **5.0×10^-5^** |
| N-Oleoylserine: Model 1 | Endocannabinoid | 1.00 | 1.20 (0.90, 1.61) | 1.39 (1.06, 1.84) | 1.69 (1.30, 2.20) | 1.14 (1.03, 1.26) | 4.0×10^-5^ |
| Model 2 |  | 1.00 | **1.23 (0.92, 1.64)** | **1.42 (1.07, 1.88)** | **1.77 (1.34, 2.32)** | **1.15 (1.03, 1.28)** | **2.2×10^-5^** |
| Linoleoyl ethanolamide: Model 1 | Endocannabinoid | 1.00 | 0.90 (0.68, 1.21) | 1.03 (0.79, 1.35) | 1.62 (1.27, 2.07) | 1.25 (1.14, 1.37) | 3.1×10^-5^ |
| Model 2 |  |  | **0.89 (0.66, 1.19)** | **1.02 (0.78, 1.34)** | **1.60 (1.24, 2.05)** | **1.25 (1.14, 1.36)** | **5.8×10^-5^** |
| Heptenedioate (C7:1-DC): Model 1 | Fatty Acid, Dicarboxylate | 1.00 | 1.10 (0.83, 1.45) | 1.47 (1.12, 1.93) | 1.71 (1.30, 2.25) | 1.28 (1.15, 1.43) | 9.6×10^-6^ |
| Model 2 |  | 1.00 | **1.07 (0.81, 1.42)** | **1.47 (1.12, 1.93)** | **1.69 (1.28, 2.22)** | **1.28 (1.15, 1.43)** | **1.2×10^-5^** |
| 13-HODE + 9-HODE: Model 1 | Fatty Acid, Monohydroxy | 1.00 | 1.07 (0.81, 1.43) | 1.34 (1.02, 1.76) | 1.70 (1.31, 2.20) | 1.28 (1.18, 1.38) | 1.1×10^-5^ |
| Model 2 |  | 1.00 | **1.10 (0.83, 1.47)** | **1.36 (1.04, 1.80)** | **1.77 (1.36, 2.30)** | **1.29 (1.19, 1.40)** | **5.1×10^-6^** |
| Glycerol 3-Phosphate: Model 1 | Glycerolipid Metabolism | 1.00 | 1.22 (0.91, 1.63) | 1.35 (1.02, 1.78) | 1.74 (1.33, 2.27) | 1.22 (1.12, 1.32) | 3.4×10^-5^ |
| Model 2 |  | 1.00 | **1.21 (0.90, 1.62)** | **1.34 (1.01, 1.78)** | **1.74 (1.32, 2.29)** | **1.22 (1.13, 1.33)** | **3.6×10^-5^** |
| Dihomolinolenate (20:3n3 or 3n6): Model 1 | Long Chain Polyunsaturated Fatty Acid (n3 and n6) | 1.00 | 1.39 (1.05, 1.84) | 1.44 (1.09, 1.92) | 1.79 (1.38, 2.32) | 1.23 (1.14, 1.34) | 1.8×10^-5^ |
| Model 2 |  | 1.00 | **1.40 (1.05, 1.87)** | **1.44 (1.08, 1.92)** | **1.79 (1.37, 2.33)** | **1.25 (1.14, 1.36)** | **3.4×10^-5^** |
| Arachidonate (20:4n6): Model 1 | Long Chain Polyunsaturated Fatty Acid (n3 and n6) | 1.00 | 1.14 (0.84, 1.54) | 1.35 (1.02, 1.80) | 2.12 (1.63, 2.77) | 1.29 (1.19, 1.40) | 1.7×10^-9^ |
| Model 2 |  | 1.00 | **1.16 (0.86, 1.58)** | **1.37 (1.03, 1.83)** | **2.18 (1.66, 2.85)** | **1.32 (1.21, 1.43)** | **1.6×10^-9^** |
| 1-Arachidonoyl-GPA (20:4): Model 1 | Lysophospholipid | 1.00 | 0.81 (0.60, 1.08) | 0.75 (0.56, 1.00) | 1.61 (1.26, 2.06) | 1.25 (1.15, 1.36) | 2.7×10^-5^ |
| Model 2 |  | 1.00 | **0.79 (0.59, 1.06)** | **0.74 (0.56, 0.99)** | **1.59 (1.24, 2.04)** | **1.25 (1.14, 1.36)** | **5.0×10^-5^** |
| 1-Palmitoyl-GPA (16:0): Model 1 | Lysophospholipid | 1.00 | 1.07 (0.80, 1.43) | 1.19 (0.90, 1.58) | 1.76 (1.37, 2.28) | 1.34 (1.23, 1.46) | 2.7×10^-6^ |
| Model 2 |  | 1.00 | **1.06 (0.79, 1.42)** | **1.17 (0.88, 1.56)** | **1.72, (1.32, 2.22)** | **1.34 (1.22, 1.46)** | **1.2×10^-5^** |
| Choline: Model 1 | Phospholipid Metabolism | 1.00 | 0.92 (0.68, 1.23) | 1.32 (0.99, 1.75) | 2.07 (1.60, 2.69) | 1.34 (1.24, 1.43) | 7.2×10^-11^ |
| Model 2 |  | 1.00 | **0.91 (0.68, 1.23)** | **1.3 (0.98, 1.74)** | **2.07 (1.60, 2.69)** | **1.33 (1.24, 1.43)** | **1.2×10^-10^** |
| Sphinganine: Model 1 | Sphingolipid Synthesis | 1.00 | 1.04 (0.77, 1.39) | 1.35 (1.02, 1.78) | 1.63 (1.26, 2.12) | 1.24 (1.15, 1.35) | 1.9×10^-5^ |
| Model 2 |  | 1.00 | **1.02 (0.76, 1.37)** | **1.31 (0.99, 1.74)** | **1.58 (1.21, 2.07)** | **1.23 (1.14, 1.34)** | **8.7×10^-5^** |
| **Nucleotides** |  |  |  |  |  |  |  |
| N6-methyladenosine: Model 1 | Purine Metabolism, Adenine containing | 1.00 | 1.00 (0.73, 1.37) | 1.30 (1.01, 1.68) | 1.64 (1.29, 2.08) | 1.30 (1.19, 1.43) | 1.4×10^-5^ |
| Model 2 |  |  | **1.01 (0.74, 1.38)** | **1.28 (0.99, 1.66)** | **1.61 (1.27, 2.05)** | **1.30 (1.18, 1.43)** | **3.4×10^-5^** |
| **Peptides** |  |  |  |  |  |  |  |
| Leucylglycine: Model 1 | Dipeptide | 1.00 | 1.27 (0.95, 1.70) | 1.21 (0.90, 1.62) | 1.85 (1.42, 2.42) | 1.31 (1.21, 1.42) | 4.9×10^-6^ |
| Model 2 |  | 1.00 | **1.28 (0.96, 1.72)** | **1.21 (0.89, 1.63)** | **1.83 (1.40, 2.40)** | **1.31 (1.21, 1.42)** | **9.7×10^-6^** |
| Valylleucine: Model 1 | Dipeptide | 1.00 | 1.11 (0.84, 1.47) | 1.08 (0.81, 1.45) | 1.83 (1.42, 2.37) | 1.28 (1.18, 1.39) | 1.8×10^-6^ |
| Model 2 |  | 1.00 | **1.11 (0.83, 1.47)** | **1.07 (0.80, 1.43)** | **1.80 (1.39, 2.33)** | **1.27 (1.18, 1.38)** | **5.0×10^-6^** |
| Glycylvaline: Model 1 | Dipeptide | 1.00 | 1.21 (0.90, 1.63) | 1.18 (0.88, 1.59) | 1.97 (1.50, 2.58) | 1.32 (1.22, 1.44) | 3.0×10^-7^ |
| Model 2 |  | 1.00 | **1.21 (0.90, 1.63)** | **1.14 (0.84, 1.54)** | **1.92 (1.46, 2.53)** | **1.31 (1.21, 1.43)** | **1.8×10^-6^** |
| ADPSGEGDFXAEGGGVR: Model 1 | Fibrinogen Cleavage Peptide | 1.00 | 0.60 (0.46, 0.77) | 0.65 (0.51, 0.83) | 0.54 (0.42, 0.70) | 0.78 (0.72, 0.84) | 5.1×10^-6^ |
| Model 2 |  | 1.00 | **0.59 (0.46, 0.76)** | **0.65 (0.51, 0.84)** | **0.54 (0.42, 0.70)** | **0.78 (0.72, 0.84)** | **9.0×10^-6^** |
| Fibrinopeptide B (1-12) : Model 1 | Fibrinogen Cleavage Peptide | 1.00 | 0.57 (0.44, 0.73) | 0.59 (0.46, 0.75) | 0.60 (0.47, 0.77) | 0.82 (0.77, 0.87) | 2.3×10^-5^ |
| Model 2 |  | 1.00 | **0.57 (0.44, 0.73)** | **0.59 (0.46, 0.76)** | **0.60 (0.47, 0.78)** | **0.82 (0.77, 0.87)** | **2.8×10^-5^** |
| Fibrinopeptide B (1-11): Model 1 | Fibrinogen Cleavage Peptide | 1.00 | 0.54 (0.42, 0.69) | 0.66 (0.52, 0.85) | 0.57 (0.45, 0.73) | 0.82 (0.77, 0.87) | 5.2×10^-5^ |
| Model 2 |  | 1.00 | **0.55 (0.43, 0.71)** | **0.65 (0.50, 0.83)** | **0.55 (0.43, 0.70)** | **0.82 (0.78, 0.88)** | **8.7×10^-6^** |
| Fibrinopeptide B (1-9): Model 1 | Fibrinogen Cleavage Peptide | 1.00 | 1.17 (0.87, 1.58) | 1.31 (0.98, 1.75) | 1.80 (1.38, 2.36) | 1.27 (1.16, 1.39) | 4.0×10^-6^ |
| Model 2 |  | 1.00 | **1.17 (0.86, 1.58)** | **1.29 (0.96, 1.74)** | **1.79 (1.35, 2.36)** | **1.26 (1.15, 1.38)** | **1.0×10^-5^** |
| Gamma-Glutamyltyrosine: Model 1 | Gamma-glutamyl Amino Acid | 1.00 | 1.22 (0.92, 1.61) | 1.13 (0.84, 1.52) | 1.74 (1.35, 2.25) | 1.29 (1.20, 1.40) | 3.1×10^-5^ |
| Model 2 |  | 1.00 | **1.21 (0.92, 1.60)** | **1.12 (0.84, 1.51)** | **1.74 (1.34, 2.26)** | **1.30 (1.20, 1.41)** | **4.1×10^-5^** |
| Gamma-Glutamyl-Alpha-Lysine: Model 1 | Gamma-glutamyl Amino Acid | 1.00 | 0.74 (0.55, 0.99) | 0.92 (0.69, 1.21) | 1.58 (1.24, 2.03) | 1.29 (1.20, 1.39) | 8.0×10^-6^ |
| Model 2 |  | 1.00 | **0.72 (0.54, 0.97)** | **0.93 (0.70, 1.23)** | **1.55 (1.21, 1.99)** | **1.29 (1.19, 1.39)** | **1.3×10^-5^** |
| Gamma-Glutamylthreonine: Model 1 | Gamma-glutamyl Amino Acid | 1.00 | 1.15 (0.86, 1.54) | 1.26 (0.94, 1.67) | 1.84 (1.42, 2.39) | 1.30 (1.21, 1.40) | 1.2×10^-6^ |
| Model 2 |  | 1.00 | **1.14 (0.86, 1.53)** | **1.23 (0.92, 1.64)** | **1.82 (1.4 0, 2.36)** | **1.30 (1.21, 1.40)** | **2.4×10^-6^** |
| Gamma-Glutamylmethionine: Model 1 | Gamma-glutamyl Amino Acid | 1.00 | 1.21 (0.91, 1.61) | 1.25 (0.95, 1.66) | 1.89 (1.46, 2.46) | 1.28 (1.18, 1.38) | 1.3×10^-6^ |
| Model 2 |  | 1.00 | **1.21 (0.91, 1.61)** | **1.24 (0.94, 1.65)** | **1.88 (1.45, 2.45)** | **1.27 (1.17, 1.38)** | **2.4×10^-6^** |
| Gamma-Glutamylisoleucine: Model 1 | Gamma-glutamyl Amino Acid | 1.00 | 1.20 (0.90, 1.62) | 1.09 (0.82, 1.46) | 1.97 (1.51, 2.58) | 1.31 (1.22, 1.41) | 5.7×10^-7^ |
| Model 2 |  | 1.00 | **1.20 (0.90, 1.62)** | **1.08 (0.80, 1.45)** | **1.99 (1.51, 2.61)** | **1.32 (1.22, 1.41)** | **1.2×10^-6^** |
| Gamma-Glutamylphenylalanine: Model 1 | Gamma-glutamyl Amino Acid | 1.00 | 1.35 (1.02, 1.81) | 1.30 (0.96, 1.75) | 2.08 (1.59, 2.71) | 1.32 (1.23, 1.42) | 8.7×10^-8^ |
| Model 2 |  | 1.00 | **1.35 (1.01, 1.80)** | **1.29 (0.95, 1.75)** | **2.04 (1.56, 2.68)** | **1.32 (1.23, 1.42)** | **2.9×10^-7^** |
| Gamma-Glutamylserine: Model 1 | Gamma-glutamyl Amino Acid | 1.00 | 1.13 (0.85, 1.52) | 1.05 (0.78, 1.42) | 1.99 (1.53, 2.59) | 1.35 (1.21, 1.49) | 1.5×10^-7^ |
| Model 2 |  | 1.00 | **1.12 (0.83, 1.50)** | **1.08 (0.80, 1.46)** | **2.00 (1.53, 2.61)** | **1.35 (1.22, 1.50)** | **9.3×10^-8^** |
| Gamma-Glutamylvaline: Model 1 | Gamma-glutamyl Amino Acid | 1.00 | 1.39 (1.04, 1.87) | 1.18 (0.88, 1.58) | 2.14 (1.64, 2.79) | 1.33 (1.24, 1.42) | 7.8×10^-8^ |
| Model 2 |  | 1.00 | **1.38 (1.03, 1.85)** | **1.16 (0.86, 1.55)** | **2.15 (1.64, 2.82)** | **1.33 (1.24, 1.43)** | **2.2×10^-7^** |
| Gamma-Glutamylleucine: Model 1 | Gamma-glutamyl Amino Acid | 1.00 | 1.31 (0.98, 1.76) | 1.17 (0.87, 1.56) | 2.14 (1.65, 2.78) | 1.31 (1.22, 1.40) | 2.4×10^-8^ |
| Model 2 |  | 1.00 | **1.31 (0.98, 1.75)** | **1.14 (0.85, 1.54)** | **2.14 (1.64, 2.80)** | **1.31 (1.22, 1.41)** | **7.8×10^-8^** |
| Gamma-Glutamylglycine: Model 1 | Gamma-glutamyl Amino Acid | 1.00 | 1.23 (0.91, 1.66) | 1.46 (1.10, 1.95) | 2.12 (1.62, 2.77) | 1.32 (1.23, 1.41) | 4.0×10^-9^ |
| Model 2 |  | 1.00 | **1.27 (0.94, 1.71)** | **1.55 (1.15, 2.09)** | **2.19 (1.66, 2.88)** | **1.32 (1.24, 1.42)** | **1.9×10^-9^** |
| Gamma-Glutamylglutamate: Model 1 | Gamma-glutamyl Amino Acid | 1.00 | 1.05 (0.78, 1.42) | 1.24 (0.93, 1.66) | 2.07 (1.60, 2.69) | 1.35 (1.26, 1.45) | 1.3×10^-9^ |
| Model 2 |  | 1.00 | **1.04 (0.77, 1.40)** | **1.25 (0.93, 1.67)** | **2.04 (1.57, 2.66)** | **1.35 (1.26, 1.45)** | **3.6×10^-9^** |
| **Xenobiotics** |  |  |  |  |  |  |  |
| S-Carboxymethyl-L-Cysteine: Model 1 | Drug - Other | 1.00 | 0.57 (0.45, 0.73) | 0.63 (0.49, 0.82) | 0.53 (0.41, 0.69) | 0.81 (0.75, 0.88) | 3.4×10^-6^ |
| Model 2 |  | 1.00 | **0.58 (0.45, 0.74)** | **0.63 (0.48, 0.81)** | **0.53 (0.41, 0.69)** | **0.82 (0.75, 0.88)** | **3.3×10^-6^** |

Abbreviations: ATBC = Alpha-Tocopherol, Beta-Carotene Cancer Prevention, BMI = body mass index, HR = hazard ratio, SD = standard deviation, SAM = S-Adenosylmethionine

^a^ Model 1: HRs and 95% CIs were estimated from Cox proportional hazards regression models adjusted for age at baseline, age at diagnosis, cancer stage at diagnosis (stage I-IV), and Gleason scores at cancer diagnosis. (data in Table 2)

^b^ Model 2: further adjusted for BMI, cigarettes smoked per day, years of smoking, serum total and serum HDL cholesterol, and calendar year of cancer diagnosis in the models.

^c^ P-value for trend was calculated by including in the regression model the ordinal value of the quartile of each metabolite and treating this as a continuous variable

**Table S2.** Hazard Ratios and 95% Confidence Intervals for the Association Between Prostate Cancer Mortality and Prediagnostic Serum Metabolites Reaching the Bonferroni Corrected Threshold Stratified by Selected Factors in the ATBC Study ^a^

| **Metabolite** | **Sub-class pathway** | **Blood collection to cancer diagnosis** | | **Cancer stage at diagnosis** | | **BMI (kg/m^2^)** | | |
| --- | --- | --- | --- | --- | --- | --- | --- | --- |
|  |  | **0-<14 y** | **≥14 y** | **Stage I and II** | **Stage III and IV** | **<25** | **25-<30** | **≥30** |
| **Amino acids and amino acid derivatives** |  | HR (95% CI) | HR (95% CI) | HR (95% CI) | HR (95% CI) | HR (95% CI) | HR (95% CI) | HR (95% CI) |
| Aspartate | Alanine and Aspartate Metabolism | 1.30 (1.18, 1.43) | 1.47 (1.26, 1.72) | 1.58 (1.39, 1.80) | 1.26 (1.14, 1.40) | 1.33 (1.16, 1.53) | 1.39 (1.24, 1.56) | 1.41 (1.10, 1.81) |
| *P for Interaction* |  | 0.11 |  | 0.03 |  | 0.62 |  |  |
| Glutamate | Glutamate Metabolism | 1.32 (1.20, 1.45) | 1.44 (1.25, 1.66) | 1.64 (1.44, 1.86) | 1.22 (1.10, 1.34) | 1.47 (1.28, 1.69) | 1.31 (1.18, 1.46) | 1.60 (1.26, 2.03) |
| *P for Interaction* |  | 0.52 |  | 0.0013 |  | 0.97 |  |  |
| Cysteine-Glutathione Disulfide | Glutathione Metabolism | 0.86 (0.79, 0.95) | 0.79 (0.68, 0.91) | 0.80 (0.71, 0.90) | 0.89 (0.81, 0.98) | 0.81 (0.70, 0.92) | 0.84 (0.75, 0.94) | 0.87 (0.73, 1.05) |
| *P for Interaction* |  | 0.72 |  | 0.19 |  | 0.90 |  |  |
| Cys-Gly, Oxidized | Glutathione Metabolism | 0.80 (0.73, 0.87) | 0.71 (0.61, 0.84) | 0.72 (0.64, 0.81) | 0.85 (0.77, 0.94) | 0.75 (0.66, 0.85) | 0.79 (0.71, 0.89) | 0.72 (0.58, 0.89) |
| *P for Interaction* |  | 0.86 |  | 0.053 |  | 0.94 |  |  |
| Cysteinylglycine | Glutathione Metabolism | 0.81 (0.74, 0.89) | 0.72 (0.63, 0.82) | 0.70 (0.62, 0.79) | 0.82 (0.74, 0.90) | 0.78 (0.69, 0.87) | 0.78 (0.69, 0.88) | 0.75 (0.61, 0.91) |
| *P for Interaction* |  | 0.97 |  | 0.085 |  | 0.79 |  |  |
| 5-Oxoproline | Glutathione Metabolism | 1.28 (1.17, 1.40) | 1.34 (1.18, 1.51) | 1.54 (1.39, 1.71) | 1.17 (1.06, 1.28) | 1.32 (1.17, 1.49) | 1.33 (1.19, 1.48) | 1.34 (1.14, 1.58) |
| *P for Interaction* |  | 0.061 |  | **0.0002**** |  | 0.94 |  |  |
| Glycine | Glycine, Serine and Threonine Metabolism | 1.18 (1.05, 1.32) | 1.45 (1.22, 1.73) | 1.23 (1.07, 1.42) | 1.23 (1.09, 1.39) | 1.26 (1.07, 1.47) | 1.24 (1.08, 1.43) | 1.57 (1.23, 1.99) |
| *P for Interaction* |  | 0.24 |  | 0.87 |  | 0.58 |  |  |
| Serine | Glycine, Serine and Threonine Metabolism | 1.25 (1.13, 1.39) | 1.59 (1.35, 1.88) | 1.48 (1.30, 1.69) | 1.24 (1.11, 1.39) | 1.36 (1.18, 1.58) | 1.35 (1.19, 1.54) | 1.40 (1.14, 1.72) |
| *P for Interaction* |  | 0.046 |  | 0.085 |  | 0.91 |  |  |
| Histidine | Histidine Metabolism | 1.17 (1.04, 1.31) | 1.25 (1.08, 1.46) | 1.35 (1.17, 1.57) | 1.11 (0.99, 1.24) | 1.31 (1.11, 1.54) | 1.19 (1.04, 1.36) | 1.41 (1.12, 1.78) |
| *P for Interaction* |  | 0.45 |  | 0.031 |  | 1.00 |  |  |
| Methionine Sulfoxide | Methionine, Cysteine, SAM and Taurine Metabolism | 1.21 (1.10, 1.34) | 1.46 (1.26, 1.70) | 1.37 (1.21, 1.55) | 1.28 (1.15, 1.42) | 1.33 (1.16, 1.53) | 1.20 (1.06, 1.36) | 1.49 (1.19, 1.86) |
| *P for Interaction* |  | 0.43 |  | 0.61 |  | 0.85 |  |  |
| Cysteine Sulfinic Acid | Methionine, Cysteine, SAM and Taurine Metabolism | 1.25 (1.14, 1.37) | 1.36 (1.20, 1.55) | 1.51 (1.35, 1.69) | 1.22 (1.11, 1.34) | 1.23 (1.09, 1.39) | 1.36 (1.21, 1.52) | 1.22 (1.02, 1.47) |
| *P for Interaction* |  | 0.45 |  | 0.024 |  | 0.84 |  |  |
| Phenylalanine | Phenylalanine Metabolism | 1.27 (1.15, 1.41) | 1.48 (1.27, 1.73) | 1.53 (1.35, 1.74) | 1.23 (1.10, 1.37) | 1.30 (1.13, 1.49) | 1.36 (1.20, 1.53) | 1.54 (1.22, 1.94) |
| *P for Interaction* |  | 0.29 |  | 0.024 |  | 0.37 |  |  |
| N-Formylphenylalanine | Tyrosine Metabolism | 1.17 (1.04, 1.32) | 1.37 (1.14, 1.66) | 1.40 (1.19, 1.65) | 1.19 (1.06, 1.35) | 1.29 (1.10, 1.51) | 1.21 (1.04, 1.40) | 1.30 (0.97, 1.74) |
| *P for Interaction* |  | 0.73 |  | 0.14 |  | 0.94 |  |  |
| Arginine | Urea cycle; Arginine and Proline Metabolism | 1.22 (1.10, 1.35) | 1.52 (1.29, 1.79) | 1.43 (1.24, 1.66) | 1.23 (1.11, 1.37) | 1.34 (1.16, 1.54) | 1.33 (1.17, 1.50) | 1.35 (1.09, 1.67) |
| *P for Interaction* |  | 0.49 |  | 0.18 |  | 0.95 |  |  |
| **Carbohydrates** |  |  |  |  |  |  |  |  |
| Erythronate | Amino Sugar Metabolism | 1.22 (1.10, 1.37) | 1.23 (1.03, 1.48) | 1.19 (1.03, 1.37) | 1.24 (1.09, 1.41) | 1.24 (1.07, 1.45) | 1.21 (1.06, 1.39) | 1.37 (1.04, 1.81) |
| *P for Interaction* |  | 0.20 |  | 0.41 |  | 0.70 |  |  |
| **Cofactors and Vitamins** |  |  |  |  |  |  |  |  |
| Oxalate (ethanedioate) | Ascorbate and Aldarate Metabolism | 0.77 (0.69, 0.86) | 0.69 (0.59, 0.80) | 0.62 (0.53, 0.71) | 0.83 (0.74, 0.93) | 0.80 (0.69, 0.93) | 0.73 (0.64, 0.83) | 0.74 (0.57, 0.97) |
| *P for Interaction* |  | 0.59 |  | 0.017 |  | 0.47 |  |  |
| Threonate | Ascorbate and Aldarate Metabolism | 0.82 (0.73, 0.91) | 0.74 (0.64, 0.87) | 0.68 (0.59, 0.78) | 0.86 (0.76, 0.96) | 0.86 (0.75, 1.00) | 0.76 (0.66, 0.86) | 0.83 (0.63, 1.10) |
| *P for Interaction* |  | 0.83 |  | 0.10 |  | 0.45 |  |  |
| **Lipids** |  |  |  |  |  |  |  |  |
| N-Stearoylserine | Endocannabinoid | 1.14 (1.02, 1.27) | 1.36 (1.15, 1.60) | 1.50 (1.25, 1.80) | 1.13 (1.00, 1.26) | 1.20 (1.01, 1.43) | 1.26 (1.09, 1.44) | 1.23 (1.00, 1.51) |
| *P for Interaction* |  | 0.39 |  | 0.0085 |  | 0.99 |  |  |
| N-Oleoylserine | Endocannabinoid | 1.10 (0.96, 1.24) | 1.19 (0.99, 1.42) | 1.37 (1.14, 1.65) | 1.07 (0.94, 1.22) | 1.15 (0.93, 1.41) | 1.18 (1.03, 1.37) | 1.11 (0.87, 1.43) |
| *P for Interaction* |  | 0.19 |  | 0.067 |  | 0.95 |  |  |
| Linoleoyl ethanolamide | Endocannabinoid | 1.23 (1.10, 1.38) | 1.26 (1.08, 1.47) | 1.46 (1.24, 1.70) | 1.16 (1.04, 1.30) | 1.28 (1.08, 1.50) | 1.27 (1.11, 1.45) | 1.30 (1.05, 1.60) |
| *P for Interaction* |  | 0.35 |  | 0.034 |  | 0.96 |  |  |
| Heptenedioate (C7:1-DC) | Fatty Acid, Dicarboxylate | 1.27 (1.11, 1.45) | 1.31 (1.09, 1.57) | 1.25 (1.06, 1.47) | 1.29 (1.13, 1.49) | 1.25 (1.05, 1.50) | 1.30 (1.11, 1.54) | 1.27 (0.99, 1.63) |
| *P for Interaction* |  | 0.27 |  | 0.51 |  | 1.00 |  |  |
| 13-HODE + 9-HODE | Fatty Acid, Monohydroxy | 1.31 (1.20, 1.44) | 1.21 (1.03, 1.41) | 1.35 (1.20, 1.52) | 1.17 (1.05, 1.31) | 1.29 (1.14, 1.47) | 1.27 (1.13, 1.43) | 1.33 (1.08, 1.65) |
| *P for Interaction* |  | 0.031 |  | 0.18 |  | 0.98 |  |  |
| Glycerol 3-Phosphate | Glycerolipid Metabolism | 1.23 (1.12, 1.36) | 1.15 (0.98, 1.34) | 1.40 (1.24, 1.60) | 1.16 (1.05, 1.29) | 1.17 (1.02, 1.35) | 1.26 (1.13, 1.42) | 1.60 (1.24, 2.05) |
| *P for Interaction* |  | 0.37 |  | 0.032 |  | 0.22 |  |  |
| Dihomolinolenate (20:3n3 or 3n6) | Long Chain Polyunsaturated Fatty Acid (n3 and n6) | 1.23 (1.11, 1.37) | 1.21 (1.05, 1.39) | 1.48 (1.28, 1.72) | 1.15 (1.03, 1.29) | 1.29 (1.08, 1.53) | 1.27 (1.13, 1.43) | 1.22 (0.99, 1.51) |
| *P for Interaction* |  | 0.36 |  | 0.001 |  | 0.84 |  |  |
| Arachidonate (20:4n6) | Long Chain Polyunsaturated Fatty Acid (n3 and n6) | 1.29 (1.16, 1.44) | 1.28 (1.12, 1.46) | 1.60 (1.38, 1.84) | 1.18 (1.06, 1.32) | 1.38 (1.17, 1.64) | 1.35 (1.19, 1.51) | 1.25 (1.04, 1.51) |
| *P for Interaction* |  | 0.071 |  | 0.019 |  | 0.48 |  |  |
| 1-Arachidonoyl-GPA (20:4) | Lysophospholipid | 1.26 (1.13, 1.41) | 1.21 (1.05, 1.38) | 1.53 (1.32, 1.77) | 1.13 (1.02, 1.25) | 1.33 (1.14, 1.56) | 1.31 (1.15, 1.49) | 1.14 (0.92, 1.41) |
| *P for Interaction* |  | 0.012 |  | 0.0016 |  | 0.52 |  |  |
| 1-Palmitoyl-GPA (16:0) | Lysophospholipid | 1.30 (1.18, 1.44) | 1.42 (1.21, 1.66) | 1.63 (1.43, 1.87) | 1.21 (1.09, 1.35) | 1.31 (1.13, 1.52) | 1.37 (1.21, 1.55) | 1.52 (1.21, 1.92) |
| *P for Interaction* |  | 0.18 |  | 0.0025 |  | 0.53 |  |  |
| Choline | Phospholipid Metabolism | 1.30 (1.19, 1.42) | 1.39 (1.23, 1.58) | 1.54 (1.38, 1.72) | 1.19 (1.08, 1.30) | 1.36 (1.21, 1.53) | 1.33 (1.20, 1.48) | 1.44 (1.20, 1.73) |
| *P for Interaction* |  | 0.40 |  | 0.0012 |  | 0.80 |  |  |
| Sphinganine | Sphingolipid Synthesis | 1.22 (1.11, 1.34) | 1.31 (1.10, 1.55) | 1.45 (1.25, 1.68) | 1.17 (1.06, 1.29) | 1.10 (0.95, 1.27) | 1.31 (1.19, 1.44) | 1.42 (1.09, 1.85) |
| *P for Interaction* |  | 0.015 |  | 0.056 |  | 0.11 |  |  |
| **Nucleotides** |  |  |  |  |  |  |  |  |
| N6-methyladenosine | Purine Metabolism, Adenine containing | 1.27 (1.14, 1.43) | 1.35 (1.15, 1.58) | 1.44 (1.24, 1.66) | 1.26 (1.11, 1.41) | 1.29 (1.11, 1.51) | 1.38 (1.20, 1.58) | 1.18 (0.92, 1.53) |
| *P for Interaction* |  | 0.053 |  | 0.23 |  | 1.00 |  |  |
| **Peptides** |  |  |  |  |  |  |  |  |
| Leucylglycine | Dipeptide | 1.26 (1.15, 1.39) | 1.43 (1.23, 1.65) | 1.57 (1.37, 1.79) | 1.23 (1.12, 1.36) | 1.40 (1.22, 1.61) | 1.34 (1.18, 1.51) | 1.26 (1.03, 1.53) |
| *P for Interaction* |  | 0.52 |  | 0.01 |  | 0.74 |  |  |
| Valylleucine | Dipeptide | 1.25 (1.14, 1.37) | 1.35 (1.16, 1.56) | 1.55 (1.36, 1.76) | 1.16 (1.04, 1.28) | 1.37 (1.20, 1.58) | 1.25 (1.11, 1.41) | 1.31 (1.07, 1.60) |
| *P for Interaction* |  | 0.21 |  | 0.00086 |  | 0.91 |  |  |
| Glycylvaline | Dipeptide | 1.28 (1.16, 1.42) | 1.40 (1.19, 1.65) | 1.58 (1.38, 1.79) | 1.25 (1.12, 1.39) | 1.29 (1.12, 1.49) | 1.39 (1.23, 1.56) | 1.34 (1.05, 1.70) |
| *P for Interaction* |  | 0.30 |  | 0.015 |  | 0.75 |  |  |
| ADPSGEGDFXAEGGGVR | Fibrinogen Cleavage Peptide | 0.81 (0.74, 0.88) | 0.70 (0.61, 0.81) | 0.64 (0.57, 0.72) | 0.85 (0.77, 0.93) | 0.76 (0.67, 0.86) | 0.80 (0.71, 0.90) | 0.69 (0.57, 0.85) |
| *P for Interaction* |  | 0.88 |  | 0.00072 |  | 0.84 |  |  |
| Fibrinopeptide B (1-12) | Fibrinogen Cleavage Peptide | 0.84 (0.78, 0.91) | 0.77 (0.68, 0.86) | 0.70 (0.63, 0.77) | 0.90 (0.83, 0.97) | 0.79 (0.70, 0.89) | 0.82 (0.75, 0.90) | 0.76 (0.65, 0.90) |
| *P for Interaction* |  | 0.76 |  | **0.00031**** |  | 0.92 |  |  |
| Fibrinopeptide B (1-11) | Fibrinogen Cleavage Peptide | 0.85 (0.79, 0.91) | 0.78 (0.70, 0.87) | 0.70 (0.64, 0.77) | 0.91 (0.84, 0.99) | 0.78 (0.70, 0.87) | 0.84 (0.77, 0.92) | 0.77 (0.66, 0.90) |
| *P for Interaction* |  | 0.82 |  | **0.0002**** |  | 1.00 |  |  |
| Fibrinopeptide B (1-9) | Fibrinogen Cleavage Peptide | 1.30 (1.16, 1.45) | 1.15 (0.98, 1.36) | 1.46 (0.27, 1.68) | 1.24 (1.10, 1.39) | 1.30 (1.12, 1.51) | 1.28 (1.12, 1.45) | 1.39 (1.05, 1.83) |
| *P for Interaction* |  | 0.15 |  | 0.097 |  | 0.85 |  |  |
| Gamma-Glutamyltyrosine | Gamma-glutamyl Amino Acid | 1.25 (1.14, 1.38) | 1.35 (1.17, 1.57) | 1.49 (1.33, 1.68) | 1.18 (1.07, 1.31) | 1.33 (1.14, 1.53) | 1.23 (1.10, 1.37) | 1.53 (1.26, 1.84) |
| *P for Interaction* |  | 0.18 |  | 0.013 |  | 0.63 |  |  |
| Gamma-Glutamyl-Alpha-Lysine | Gamma-glutamyl Amino Acid | 1.24 (1.14, 1.36) | 1.36 (1.20, 1.55) | 1.49 (1.34, 1.66) | 1.20 (1.09, 1.33) | 1.37 (1.21, 1.56) | 1.23 (1.10, 1.38) | 1.40 (1.19, 1.66) |
| *P for Interaction* |  | 0.69 |  | 0.0073 |  | 0.99 |  |  |
| Gamma-Glutamylthreonine | Gamma-glutamyl Amino Acid | 1.24 (1.13, 1.36) | 1.43 (1.25, 1.63) | 1.54 (1.37, 1.72) | 1.17 (1.06, 1.29) | 1.42 (1.23, 1.63) | 1.25 (1.13, 1.39) | 1.38 (1.16, 1.65) |
| *P for Interaction* |  | 0.11 |  | 0.0014 |  | 0.93 |  |  |
| Gamma-Glutamylmethionine | Gamma-glutamyl Amino Acid | 1.21 (1.11, 1.33) | 1.46 (1.24, 1.71) | 1.47 (1.30, 1.66) | 1.21 (1.09, 1.33) | 1.38 (1.19, 1.61) | 1.21 (1.09, 1.35) | 1.48 (1.21, 1.81) |
| *P for Interaction* |  | 0.59 |  | 0.038 |  | 0.85 |  |  |
| Gamma-Glutamylisoleucine | Gamma-glutamyl Amino Acid | 1.28 (1.18, 1.39) | 1.40 (1.22, 1.60) | 1.57 (1.41, 1.74) | 1.17 (1.07, 1.28) | 1.42 (1.25, 1.60) | 1.27 (1.15, 1.40) | 1.39 (1.15, 1.69) |
| *P for Interaction* |  | 0.41 |  | **0.00023**** |  | 0.97 |  |  |
| Gamma-Glutamylphenylalanine | Gamma-glutamyl Amino Acid | 1.27 (1.17, 1.39) | 1.41 (1.24, 1.61) | 1.52 (1.37, 1.69) | 1.20 (1.09, 1.31) | 1.39 (1.21, 1.59) | 1.30 (1.18, 1.44) | 1.38 (1.16, 1.64) |
| *P for Interaction* |  | 0.27 |  | 0.0037 |  | 1.00 |  |  |
| Gamma-Glutamylserine | Gamma-glutamyl Amino Acid | 1.33 (1.18, 1.51) | 1.33 (1.10, 1.60) | 1.73 (1.47, 2.05) | 1.21 (1.07, 1.37) | 1.47 (1.21, 1.80) | 1.38 (1.19, 1.60) | 1.18 (0.95, 1.48) |
| *P for Interaction* |  | 0.038 |  | 0.0024 |  | 0.61 |  |  |
| Gamma-Glutamylvaline | Gamma-glutamyl Amino Acid | 1.28 (1.18, 1.39) | 1.45 (1.27, 1.65) | 1.57 (1.41, 1.74) | 1.19 (1.08, 1.30) | 1.42 (1.25, 1.61) | 1.28 (1.16, 1.42) | 1.42 (1.17, 1.71) |
| *P for Interaction* |  | 0.39 |  | 0.00041 |  | 1.00 |  |  |
| Gamma-Glutamylleucine | Gamma-glutamyl Amino Acid | 1.26 (1.16, 1.37) | 1.41 (1.24, 1.59) | 1.55 (1.40, 1.72) | 1.18 (1.07, 1.29) | 1.41 (1.24, 1.59) | 1.27 (1.15, 1.40) | 1.39 (1.16, 1.66) |
| *P for Interaction* |  | 0.34 |  | **0.00032**** |  | 0.98 |  |  |
| Gamma-Glutamylglycine | Gamma-glutamyl Amino Acid | 1.27 (1.17, 1.38) | 1.43 (1.26, 1.64) | 1.53 (1.38, 1.71) | 1.21 (1.11, 1.33) | 1.43 (1.26, 1.62) | 1.27 (1.15, 1.40) | 1.42 (1.20, 1.69) |
| *P for Interaction* |  | 0.41 |  | 0.0032 |  | 0.99 |  |  |
| Gamma-Glutamylglutamate | Gamma-glutamyl Amino Acid | 1.31 (1.20, 1.42) | 1.42 (1.25, 1.62) | 1.61 (1.44, 1.80) | 1.23 (1.12, 1.35) | 1.48 (1.30, 1.69) | 1.28 (1.16, 1.41) | 1.51 (1.26, 1.82) |
| *P for Interaction* |  | 0.28 |  | 0.0012 |  | 1.00 |  |  |
| **Xenobiotics** |  |  |  |  |  |  |  |  |
| S-Carboxymethyl-L-Cysteine | Drug - Other | 0.85 (0.77, 0.93) | 0.72 (0.63, 0.82) | 0.70 (0.62, 0.79) | 0.87 (0.78, 0.96) | 0.82 (0.72, 0.94) | 0.76 (0.69, 0.85) | 0.84 (0.68, 1.03) |
| *P for Interaction* |  | 0.71 |  | 0.023 |  | 0.80 |  |  |

Abbreviations: ATBC = Alpha-Tocopherol, Beta-Carotene Cancer Prevention, HR = hazard ratio, SAM = S-Adenosylmethionine

^a^ HRs and 95% CIs were estimated from Cox proportional hazards regression models adjusted for age at baseline, age at diagnosis, cancer stage at diagnosis (stage I-IV), and Gleason scores at cancer diagnosis. Bonferroni corrected threshold: 0.05/(49×3)=3.4×10^-4^

**Table S3.** Hazard Ratios and 95% Confidence Intervals for Serum Metabolites Achieving the Bonferroni Corrected Threshold Using Stepwise Cox Proportional Hazards Regression Analysis in the ATBC Study ^a^

|  | HRs (95% CIs) | | | | |
| --- | --- | --- | --- | --- | --- |
|  | Step 1 | Step 2 | Step 3 | Step 4 | Step 5 |
| Gamma-Glutamylglutamate | 1.35 (1.26, 1.45) | 1.34 (1.25, 1.44) | 1.26 (1.15, 1.37) | 1.19 (1.08, 1.31) | 1.26 (1.12, 1.41) |
| Heptenedioate (C7:1-DC) |  | 1.25 (1.13, 1.39) | 1.25 (1.13, 1.39) | 1.25 (1.12, 1.38) | 1.25 (1.13, 1.39) |
| Oxalate (ethanedioate) |  |  | 0.87 (0.79, 0.97) | 0.87 (0.79, 0.97) | 0.88 (0.79, 0.98) |
| Arachidonate (20:4n6) |  |  |  | 1.12 (1.01, 1.23) | 1.17 (1.05, 1.31) |
| Linoleoyl ethanolamide |  |  |  |  | 0.88 (0.78, 0.99) |

Abbreviations: HR = hazard ratio, CI = confidence interval

^a^ HRs and 95% CIs were calculated using stepwise Cox proportional hazards regression models for metabolites achieving the Bonferroni corrected threshold (P-values <5.2×10^-5^), and adjusted for age at baseline, age at diagnosis, cancer stage at diagnosis (stage I-IV), and Gleason scores at cancer diagnosis.
